# An investigation of English language teachers’ motivation from an ecological perspective: A case study from mainland China

**DOI:** 10.1371/journal.pone.0321139

**Published:** 2025-04-29

**Authors:** Xueshan Zhang

**Affiliations:** English Language Center, Beijing Normal University-Hong Kong Baptist University (BNBU), Zhuhai, Guangdong Province, China; Ahvaz Jundishapur University: Ahvaz Jondishapour University of Medical Sciences, IRAN, ISLAMIC REPUBLIC OF

## Abstract

Language teachers’ teaching motivation remains an under-researched area. To address this research gap, this qualitative study explored dynamic changes of English teachers’ teaching motivation in a Chinese public high school by utilizing possible selves theory from an ecological perspective, which is a comprehensive approach that highlights teachers’ agency, values their unique experiences and their interactions with the spatial-temporal context. The concept of possible selves encompassing three distinct aspects: the actual self reflects current self-perception; the ideal self embodies future aspirations, and the ought-to self denotes perceived obligations and duties. Data were collected through semi-structured interviews, teachers’ reflective journals, the researcher’s journal, and various documents (i.e., online information provided by the official website of the local high school and its official WeChat account). Against the backdrop of a plethora of research focusing on the more individualistic ideal self, this study found the prominent role of the ought-to self on teachers’ teaching motivation development because participants strongly internalized the ought-to self. In addition, most of their ideal images were in alignment with the ought-to self. This agreement between ideal selves and the ought-to self enhanced their motivation. The article concluded by suggesting that the concept of possible selves should be utilized in teacher education program design to understand and support teachers’ unique aspirations and obligations.

## Introduction

Studies of second and foreign language learning motivation (henceforth, L2 learner motivation) have moved toward a more situated and comprehensive approach [1], recognizing that fluctuations in learners’ motivation relate to a constellation of external and internal variables, including beliefs, knowledge, self-perceptions, and emotions [2]. Considering the scantiness of research on foreign language teachers’ teaching motivation [3] and the latest development in L2 learner motivation, this study explores the evolution of English teachers’ teaching motivation in a Northern Chinese state high school through the lens of their possible selves evolution (i.e., ideal self, actual self, and ought-to self) from an ecological perspective. The innovative contribution of this study lies in its emphasis on the prominent role of the ought-to self and its relationship with teachers’ ideal selves, which challenges prior research that illuminates ideal selves while almost ignores the ought-to self. Furthermore, from the ecological perspective, this study leverages a more holistic and dynamic approach to explore English teachers’ teaching motivation, perceiving teachers as thinking and feeling individuals with unique histories and backgrounds and examining the interplay between teachers and the fluid micro-and macro-contexts in which they operate. Finally, this study significantly contributes to the existing literature on teacher motivation by providing empirical evidence on the evolution of teaching motivation among high school English teachers in China, a context that has been relatively underexplored. Utilizing the lens of possible selves theory, this research not only deepens our understanding of teacher motivation within this unique cultural context, but also broadens the applicability of possible selves theory in educational research.

### 1.1. The concept of teachers’ teaching motivation

The word “motivation” originates from the Latin verb *movere*, which means to move [4]. It has been generally understood as “a drive that moves people to do something by nature” [5, p.3]. However, due to the complex and multidimensional nature of motivation as a construct, there has been little agreement on a single and universal definition among researchers with different theoretical commitments [6,7].

While existing definitions provide insights into teachers’ teaching motivation, they tend to overlook its dynamic nature and the interactions between teachers and multilayered contextual factors. This study, therefore, defines teaching motivation as the complex and dynamic motives that drive teachers to persist in the profession, shaped by their experiences, cognition, emotions, and interaction with context. In essence, teacher motivation is inextricably tied to both internal and external factors related to human beings and their intricate lives [2].

### 1.2 Possible selves theory

According to Markus and Nurius [8], possible selves embody an individual’s conceptions of their potential future states and establish a conceptual bridge between cognitive processes and motivation. Higgins [9] extended the construct of possible selves by defining three distinct constructs: actual self, ideal self, and ought-to self. The actual self signifies one’s present self-concept, reflecting the attributes they believe they possess. The ideal self embodies future aspirations, representing desired qualities. The ought-to self pertains to duties and responsibilities, denoting traits individuals feel obligated to have. The theory of possible selves also posits that people’s ability to image allows them to visualize these various selves and thereby exceed the confines of their current reality [10]. This process of imagination has concrete effects on our current experiences, demonstrating the substantial motivational influence of our perceptions of success and failure.

Contrasting with other motivation theories such as self-determination theory, which primarily concentrate on teachers’ current experiences and knowledge, possible selves are tied to teachers’ future expectations, acting as motivational assets. Despite being future-focused, possible selves are intimately linked with individuals’ past and present selves.

### 1.3 Possible selves theory in L2 teacher motivation research

In the field of applied linguistics, Dörnyei [11] pioneered the L2 Motivation Self System, drawing on possible selves theories, to delve into learners’ motivation for studying foreign languages. Kubanyiova [10,12] subsequently developed the Possible Language Teacher Self model built on Dörnyei’s (2005) framework. This model was designed to investigate changes in the conception of teachers in a professional development program.

The Possible Language Teacher Self model comprises the ideal language teacher self (i.e., ideal teaching selves), the ought-to language teacher self (i.e., teachers’ cognitive representation of their responsibilities and obligations, normative pressure from school regulations, and students’ and colleagues’ expectations), and the feared language teacher self (i.e., images that teachers do not want to become). Kubanyiova [12] underscored the centrality of teachers’ ideal selves to their teaching motivation, leading to self-regulatory efforts to minimize discrepancies between actual and ideal teaching selves. However, this emphasis on the ideal self may overlook the potential motivational influence of the feared self and the ought-to self, a gap that future research could address.

Given the expansion of the possible selves theory in applied linguistics, a few studies on teachers’ motivation have started to exploit this powerful, multifaceted, comprehensive, and inclusive theoretical lens for their analysis. Costa [13] offered a pioneering exploration into the evolution of possible selves and their impact on the motivation of novice English teachers in a Brazilian elementary public school. This study underscored the dual role of actual selves: positive actual selves bolster teachers’ self-efficacy and motivation, while negative ones can either stimulate or impede progress. This study also unveiled that positive future images may stimulate teachers’ motivation, while feared selves play critical roles in completing teachers’ daily tasks. This nuanced understanding of the actual self’s impact on motivation sets a foundation for further research into the dynamics of teacher motivation.

Kumazawa’s [14] study, conducted in Japan, further contributed to this line of inquiry by shifting the focus from ideal selves and actual selves to the discrepancies between ideal selves and teachers’ actual and ought-to selves. The conclusion that these discrepancies serve as a severe demotivator underscored the importance of considering the dynamic interplay among different facets of the self in understanding teachers’ motivation.

More recently, Sahakyan [15] expanded the scope of possible selves theory in L2 motivation studies by examining the experiences of five teachers at the initial stage of their careers at three universities in Armenia. The finding that these teachers’ unrealistic ideal selves changed due to the constraints of multilayered contextual factors highlights the importance of considering the impact of contextual factors on the formation and evolution of possible selves. Furthermore, the concept of feasible selves, which fits well with the local context and comprises components of ideal, ought-to, and feared selves, opens up new avenues for future research. However, given the context-sensitive nature of possible selves, it’s uncertain whether this concept is universally applicable.

Notably, the application of the possible selves theory in studying teacher motivation is still in its infancy, necessitating further research to validate its explanatory power. Furthermore, given that most research, except for Sahakyan’s [15] study, focuses solely on early-service teachers, there is a pressing need for additional research attention on teachers at various career stages. Additionally, existing research has largely overlooked Chinese English teachers, particularly in examining the unique characteristics of their possible selves’ evolution and its consequential impact on their teaching motivation. This study aims to address these research gaps by investigating the influence of English teachers’ possible selves on their motivational trajectory throughout their teaching career at various stages, within a Chinese state high school setting. In doing so, it seeks to illuminate underexplored aspects of teacher motivation, significantly enriching the existing body of knowledge and deepening our understanding of this phenomenon within the Chinese context.

### 1.4 Combining possible selves theory with an ecological perspective

Despite the versatile and explanatory power of the possible selves theory, it has been criticized for its overreliance on ego-centric constructs [16]. To address this limitation, this study intends to explore trajectories of teachers’ possible selves from an ecological perspective, aiming to investigate and understand the inextricable relations between various internal and external components.

The term “ecology” was created by the German biologist Ernst Haeckel [17] to refer to “the study of the relationships between all the various organisms and their physical environment” [18, p. 114]. Thus, ecology research differs from traditional scientific research and the reductive perspective, which controls the environment experimentally and investigates smaller pieces of a complex phenomenon.

An ecological perspective in second language acquisition (SLA) research, as explained by Kramsch and Steffensen [19], emphasizes holism. Kramsch [20] further elucidates that ecology allows researchers to account for phenomena that might otherwise go unnoticed. This perspective underscores interconnectedness, where every part is linked to each other and the whole, and interdependence, where changes in one part cause shifts in others. It also highlights the multidirectional interactions between different linguistic phenomena [20,21]. Moreover, an ecological perspective appreciates diversity, emphasizing the unique and specific [22,23]. Furthering this idea, Barkhuizen & Consoli [24] suggest that from an ecological viewpoint, humans are perceived as living organisms with their unique life stories and idiosyncrasies.

From an ecological perspective, this study adopts a holistic approach emphasizing teachers’ agency, valuing their experiences, feelings, and idiosyncrasies, and taking into account how their possible selves influence and are influenced by the spatial-temporal context environment in which they live.

### 1.5 Research goals

Despite the foundational understanding of language teacher motivation provided by existing studies, there remains a need for further research employing the possible selves theory, particularly focusing on the evolution of teachers’ possible selves and their impact on teaching motivation across diverse contexts. Addressing the identified research need, this study delves into the role of possible selves in shaping the motivational trajectory of English teachers at various career stages within a Chinese state high school setting. It aims to uncover the unique characteristics of these teachers’ possible selves and assess their influence on motivational levels. Another crucial aspect of this research is to explore the interplay between teachers’ possible selves and their environmental factors, investigating how these dynamics further impact their motivation. Through this inquiry, the study endeavors to deepen the understanding of teacher motivation, with a specific focus on the Chinese context. The study was driven by the following question:

1.How can changes in teachers’ motivation be described through the lens of possible teacher selves?
2.How do the environmental factors interact with the evolution of teachers’ possible selves?

## The study

### 2.1 The research stance

This study seeks to evaluate teachers’ motivation through their personal interpretation of their work experiences, acknowledging the subjective meanings formed by their diverse social and historical influences [25]. As Merriam and Tisdell [26] suggest, a single phenomenon can have multiple realities due to individuals’ differing perceptions. Aligning with this view, the researcher adopts an interpretivist paradigm as the philosophical foundation for this study, recognizing the multiplicity of realities in the exploration of teachers’ motivation.

Paltridge and Phakiti [27] assert that within the interpretivist paradigm, realities are not singular and objective but are multiple and subjective, existing within individuals’ minds. This stance, at the ontological level, refers to the belief in multiple realities that are constructed rather than discovered, contrasting with the idea of a single, observable reality [26]. When it comes to the epistemological level, which concerns the nature and scope of knowledge, the interpretivist paradigm assumes that knowledge is obtained through participants’ personal understandings and interpretations of their experiences and surroundings [28].

According to Hitchcock and Hughes [29], ontological assumptions give rise to epistemological assumptions, finally giving rise to methodological considerations. Therefore, it is essential to note that after establishing its philosophical stance, this study adopts the qualitative approach, which aims to understand “how people understand their experiences, how they construct their worlds, and what meaning they contribute to their experiences” [26, p. 3].

### 2.2 A multiple case study approach

Given the philosophical stance and aim of this study, a multiple case study approach was deemed most suitable. This approach allows for a holistic exploration of complex phenomena, as opposed to a reductionist view [30]. Furthermore, case studies, characterized by boundness, natural context, and in-depth analysis [31], facilitate a clearer understanding of research results by presenting real experiences in context, rather than relying on abstract variables and numerical analysis [28].

By adopting this approach, the study can holistically assess teachers’ motivation, elucidating the formation and evolution of in-service high school English teachers’ possible selves through lived experiences. Moreover, this approach allows for the amplification of teachers’ voices, potentially impacting decisions of educators, policymakers, and school leaders. Ultimately, insight into teachers’ lived experiences can foster a better understanding among students and parents of teachers’ circumstances and efforts.

### 2.3 Research context

In China, where a long-standing tradition of “respecting education highly” [32, p. 8] exists, the Confucian belief prevails that education can transform individuals and nations [33]. This reverence for education, coupled with its role as a socioeconomic ladder [34], offers teachers job stability and secure pension plans [35,36], contrasting with high turnover rates in Western countries.

Particularly, English Language Education is seen as a crucial tool for China’s globalization [33,37] and a form of social capital for individuals, linked with economic benefits and social prestige [38,39]. In response, the subject of English holds significant weight in the high stake and knowledge-based university entrance exam [40,41]. Consequently, grammar-oriented English classes have been predominant for many years in high school [42,43], putting significant pressure on teachers to boost test scores due to high expectations from administrators and parents [44,45].

Narrowing the focus to the study’s micro context, a high school located in a small county in northern China, it had been ranked below medium among county public high schools. However, recent managerial reforms have led to a rise in its ranking. Notably, local education management has seen significant improvements, such as policies to attract excellent students locally, and the school’s management has become more transparent and fairer.

### 2.4 The participants

At the initial stage of this study, a mixture of convenience and purposeful sampling methods was adopted as access to the local high school (the research setting) was obtained through a local gatekeeper, one of the researcher’s university classmates. After identifying the group of English teachers in this high school as potential participants, a purposive sample was utilized to seek core participants capable of providing “rich and diverse insights into the phenomenon” [46, p. 126].

In order to construct a representative and diverse sample, the researcher set forth explicit criteria for participant selection. These criteria included factors of age, years of teaching experience, gender, and the categories of student groups they were instructing at the time of the research. This was intended to capture a wide range of experiences and perspectives within the teaching profession. For instance, the age and years of teaching experience criteria were designed to include both novice and experienced teachers, thus capturing different stages of career development. The gender criterion was set to ensure representation from both male and female teachers, acknowledging the potential influence of gender on teaching styles and experiences. Lastly, the categories of student groups being taught by the participants were considered to account for potential variations in teaching methods and challenges faced across different student demographics. In this way, despite the small sample size, the researcher aimed to encapsulate the broader population of English teachers through a comprehensive and diverse set of selection criteria.

Considering the heterogeneous sample, the multiple data collection instruments, the limitation of funds and time available for a doctorate study, eight participants were recruited initially. However, due to work commitments, one participant withdrew after the second interview, leaving seven in-service English teachers (Cali, Wynne, Jack, Sophia, Lisa, Lily, and Harley—all pseudonyms) who completed the data collection phase from March to September 2022 at the local public high school. Table 1 introduces detailed background information of participants.

**Table 1:** Background Information of Participants.

Participants	Gender	Age	Qualifications	Years of Teaching Experience	Current Teaching Group
**Jack**	Male	41	BA in English	17	Grade One
**Cali**	Female	26	BA in English	3	Grade Two
**Harley**	Female	34	Normal University (offering four-year teacher education programs)/English Major	8	Grade Two
**Wynne**	Female	38	Normal College (offering three-year teacher education programs)/English Major	18	Grade Two
**Sophia**	Female	40	Normal University/English Major	17	Grade Two
**Lily**	Female	43	Normal University/English Major	23	Grade Two
**Lisa**	Female	51	Normal University/English Major	28	Grade Two

### 2.5 Ethical consideration

In adherence to the ethical guidelines delineated by Patton [47], this study was conscientiously designed to prioritize the interests of the participants. The ethical protocol for research involving human subjects, stipulated by the Hong Kong Polytechnic University, was meticulously followed, and the study received the requisite ethical approval (HSEARS20220318007). Comprehensive details of the project were disclosed to the participants, emphasizing their right to voluntary participation, and their informed consents were duly obtained. Additionally, stringent measures were implemented to ensure the confidentiality of the data gathered. The study also addressed critical ethical considerations such as anonymity and the dynamics of the participant-researcher relationship. Anonymity was scrupulously maintained through the use of pseudonyms and secure data storage methods. Concurrently, the researcher endeavored to foster a relationship of trust and rapport with the participants, allowing them the space to express their opinions freely. The researcher also demonstrated sensitivity towards the emotions of the participants and extended assistance as necessitated.

### 2.6 Data collection

The study, conducted from March 28 to October 28, 2022, utilized semi-structured interviews based on the Teacher Interview Guide (Appendix A) as the primary data collection tool. It was split into two phases due to the summer break. The first phase, from end of March to mid-July, aimed to gather data on participants’ backgrounds, teachers’ evolving possible selves, and their interactions with various factors. The second phase in September was for meaning-checking, ensuring accurate analysis of teachers’ experiences.

Over the six-month study period, due to the Covid-19 pandemic, all 28 interviews were conducted online, each lasting around 50 minutes. Exceptions included extended sessions with Lily (96 minutes), Sophia (93 minutes), and Harley (86 minutes). Participants, interviewed three to five times depending on their availability, chose Chinese for accurate depiction of their experiences and motivational evolution. Another complementary data collection tool designed to enhances the validity of research findings was teachers’ reflective journals, which were submitted weekly after the first interview. Multiple data sources enabled the researcher to understand the same problem from different standpoints and enrich the findings. For example, teachers mentioned their teaching methods and beliefs during the interviews and highlighted the same information in their reflective journals. Table 2 illustrates details of data collection for this study. In this table, interview information shows the duration and the date of each interview; reflective journal information shows the number of reflective journals received.

**Table 2:** Details of the Data Collection.

Participants	Tasks	Phase One				Phase Two
		April	May	June	July	September
**Cali**	Interview	55min (6^th^)	50min (9^th^)	41min (17^th^)	0	25min (20^th^)
	Reflective Journal	2	4	4	1	2
**Sophia**	Interview	60min (6^th^)	50min (7^th^)	64min (17^th^)	93min (9^th^)	0
	Reflective Journal	2	5	3	1	0
**Lily**	Interview	50min (7^th^)	54min (7^th^)	0	50min (2^nd^)	0
	Reflective Journal	2	5	3	1	0
**Wynne**	Interview	56min (7^th^)	46min (9^th^)	47min (17^th^)	22min (16^th^)	0
	Reflective Journal	3	4	3	1	2
**Lisa**	Interview	52min (7^th^)	51min (13^th^)	44min (17^th^)	0	10min (22^nd^)
	Reflective Journal	2	4	3	1	0
**Jack**	Interview	55min (8^th^)	55min (7^th^)	65min (23^rd^)	31min (7^th^)	12min (20^th^)
	Reflective Journal	2	4	3	1	0
**Harley**	Interview	53min (8^th^)	62min (28^th^)	86min (28^th^)	0	35min (13^th^)
	Reflective Journal	2	1	4	2	0

Upon the conclusion of each interview, the researcher embarked on a meticulous process of data immersion. This entailed multiple iterations of listening to the audio recordings, transcribing them into Word documents, and verifying the accuracy of the transcriptions. An initial analysis of the transcripts was conducted to identify areas requiring further elucidation. In addition to this preliminary analysis of interview transcripts, a careful reading of teachers’ reflective journals was helpful for the researcher to determine important questions that needed further clarification and more in-depth answers.

Starting from the third interview, participants were engaged in a process of member checking, where they were requested to review preliminary interpretations of their ‘possible selves’ to ensure the accuracy of the researcher’s understanding, thereby facilitating immediate rectification of any discrepancies. Prior to the concluding interviews, each participant was presented with a comprehensive visual representation of their data analysis results (both Chinese and English version), organized according to identified superordinate codes and themes pertinent to each research question, for their validation.

### 2.7 Data analysis

This study employed thematic analysis, a method that facilitates the identification, analysis, and coding of emergent themes within data, offering accessibility and flexibility [48,49]. The analysis began with an immersive process [50], involving repeated listening to the interviews, transcription, and translation from Chinese to English, utilizing a repeated checking strategy to ensure the fidelity of participants’ meanings. Participants were sent both versions of their transcripts to verify accuracy. Supplementary data, including reflective journals and related documents, were also translated from Chinese to English.

**Fig 1:**
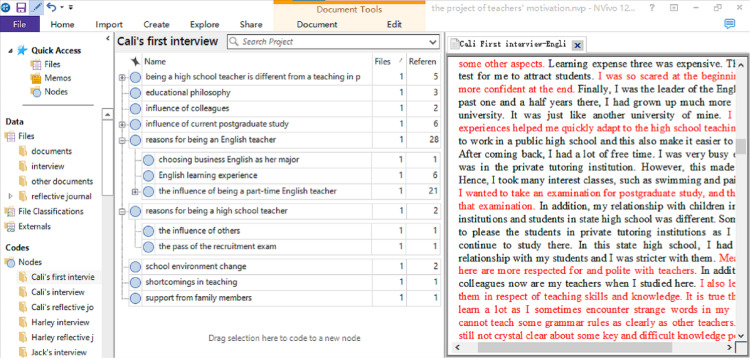
The Codes of Cali’s First Interview.

**Fig 2:**
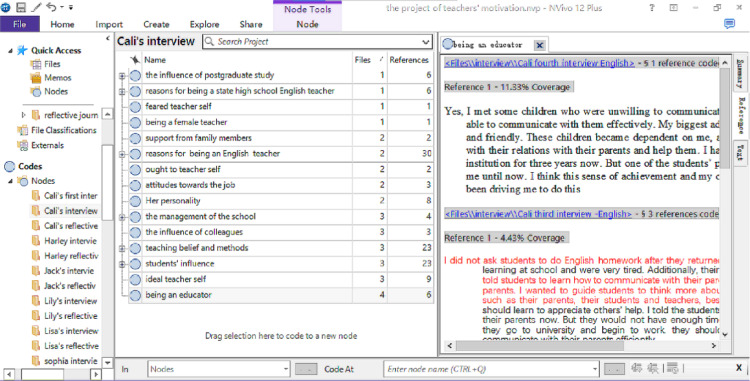
The Overarching Codes of Cali’s Interviews.

**Fig 3:**
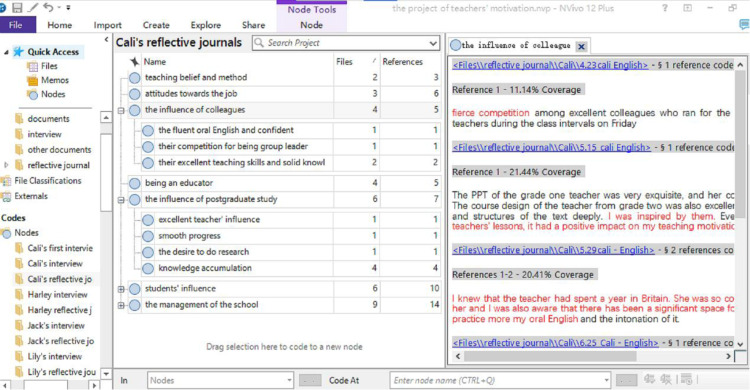
The Overarching Codes of Cali’s Reflective Journals.

Utilizing Nvivo 12 software, the researcher efficiently organized, managed, and coded a substantial data set, comprising 30 hours of interview transcripts and 75 reflective journals. An initial list of codes was identified by meticulously analyzing the transcripts and journals with Nvivo 12. As new data emerged from subsequent interviews, overarching codes were determined and organized using the same software. These codes were then systematically categorized into themes pertinent to each research question through a reflective, iterative process. Participants were presented with comprehensive diagrams of their respective data analyses for verification. Figs 1–3, screenshots from Nvivo 12 during the coding process, depict the coding of Cali’s first interview, the overarching codes from Cali’s interviews and reflective journals.

## Results

As prominently highlighted by the data analysis, the influence of the ought-to self emerges as a significant factor among the participants and shares a close relationship with their ideal selves. Consequently, the ensuing discussion aims to provide an in-depth analysis of the impact of the ought-to teacher self on teaching motivation and its interaction with environmental influences. Further discussion will be dedicated to the exploration of the relationship between the ought-to teacher self and participants’ ideal selves, and their collective influence on teaching motivation.

### 3.1 The prominent role of the ought-to teacher self

Given the importance of English as a core subject in university entrance exams, high school principals and parents place significant emphasis on students’ English exam scores. Consequently, all participants strive to realize their ought-to teacher self: a teacher capable of improving students’ English exam scores. The strongly internalized nature of this ought-to teacher self was a notable characteristic across all participant data. For instance, Lily expressed that her students’ high grades hold more value to her than any personal awards.

*I thought I achieved my purpose if my students’ grades are good and they can be accepted by their ideal universities (Lily’s Interview 2).*

Teachers deeply internalize their ought-to teacher self due to societal and administrative expectations. Additionally, participants hold the belief that excellent exam performance is the sole path to a promising future for students in this small county.

*Students in backward areas have no other choice but to go to university to have a bright future. The university entrance examination can change their fate. (Lily’s Interview 2)*

This belief stems from teachers’ personal experiences. Most originated from small villages and farming or working families, and through their own educational endeavors, they were able to elevate their social status and improve their living conditions.

The prominent role of the ought-to teacher self has increased in recent years because of changes in the educational environment in the county and school management. Previously, the local high school primarily admitted underperforming students, leaving teachers feeling demotivated due to limited improvement prospects. However, the situation improved significantly with the efforts of two successive education bureau directors, attracting higher-quality students and boosting teachers’ confidence in aiding academic improvement. This led to a positive cycle, elevating teachers’ morale and the school’s academic ranking.

*It is also because of the two leaders of the education bureau, who did a lot of work to reverse the situation of losing excellent students and retain most of the high-quality students in our school. (Wynne’s Interview 3)*

To enhance school management, the former principal introduced a quantified system for teacher performance evaluation. This system established clear rules and standards, scoring teachers based on their work performance, which was crucial for their awards and promotions. Notably, students’ academic performance constituted 60% of a teacher’s overall score. The school’s emphasis on students’ grades was primarily due to local inter-school competition. The continuous and significant improvement in the school’s ranking, a result of concerted efforts by management and teachers, fostered a sense of collective pride and invigorated their professional enthusiasm.

*Teachers are very clear about their goals, such as the rate of students enrolled at normal as well as top universities. Teachers are highly motivated. They all try their best to achieve those clear goals. Everyone emphasizes students’ grades. (Wynne’s Interview 4)*

Moreover, the principal’s significant transformation of the work environment and increased attention to teachers spurred their contribution to school development. Meanwhile, the school launched various teaching research initiatives, like joint lesson planning and co-testing with students, to aid teachers in aligning with the ought-to self.

*In recent years, our head master has done a lot of work to improve our working enthusiasm. Now we are in high spirits and full of energy. (Harley’s Reflective Journal, April 24)*

In conclusion, the participants’ deeply internalized ought-to self underscores its pivotal role in teachers’ motivation, shaped by personal beliefs, experiences, and school context factors.

### 3.2 Ideal teacher self: A dynamically stable evolution being influenced significantly by the ought-to self

Lisa, unique among participants, lacked ideal images and only motivated the ought-to self, belong to the non-visionary type mentioned by Rahmati et al. [51]. Initially detached and uninterested in student learning, she faced challenges when failing to meet the ought-to self requirements. Hindered by lack of external support and her introverted nature, Lisa struggled with self-improvement. Contrastingly, other participants, identified by Rahmati et al. [51] as the visionary type, meaning teachers with a clear articulation of future teacher images, held clear ideal images with stable evolution, which were significantly influenced by their ought-to teacher self. Jack, for example, evolved his ideal teacher self from simply explaining knowledge points clearly to being able to answer all students’ questions, aligning with the goal of enhancing students’ exam scores. With improved management and professional development, Jack overcame early career hurdles, teaching A-level students and taking on leadership roles. This led him to envision a new ideal self: a teacher adept at boosting high-achieving students’ English scores. Despite this focus potentially hindering long-term student development, Jack prioritized students’ academic performance due to its impact on his career progression and income. This highlights the strong alignment between Jack’s ideal self evolution and the obligations of the ought-to self.

*Students’ enrollment by universities is based on their grades. Therefore, my focus is on students’ grades. I work for and think about students’ grade. I’m worried that I don’t pay attention to students’ moral education and future growth (Jack’s Reflective Journal, July 6)*

Lily’s ideal teacher self evolved from being a knowledge provider to a mentor aiding students in knowledge application for exams, and ultimately to an innovator using creative teaching methods for easy comprehension of teaching content. While the first two versions of her ideal selves were closely aligned with the ought-to self, focusing on students’ grades, the last version also considered easing students’ stress and promoting their mental well-being, demonstrating a partial alignment with the ought-to self.

Like Jack and Lily, the ought-to self was also pivotal in shaping the ideal selves of other participants. The alignment of their ideal selves with the demands of the ‘ought-to’ self enhances their teaching motivation.

## Discussion

### 4.1 The central role of the ought-to self

While past research has primarily focused on the role of ideal teacher selves in motivation [15,52], this study brings a nuanced perspective by highlighting the influential roles of both the ought-to self and ideal self in teaching motivation. This stands in contrast to Hiver’s [52] study, which downplayed the impact of normative obligations on motivation, and Kumazawa’s [14] findings that participants seldom integrate the ought-to self into their ideal selves. Instead, this study underscores a strong internalization of the ought-to self, thereby redefining its motivational importance.

Ryan and Deci [53] delineate four facets of extrinsic motivation that elucidate participants’ strong internalization of the ought-to self. Firstly, failing to meet the ought-to self’s expectations risks job loss and shame. Secondly, participants feel guilty if they do not fully aid students in improving exam scores. Thirdly, participants universally acknowledge the significant impact of students’ exam scores on their futures, particularly in the context of escalating educational competition. High exam performance enhances students’ chances of acceptance into top universities, thereby leading to elevated social status and job remuneration. This is particularly pertinent in China, where young people are seen as extensions of their families, with their development linked to their family’s standing [54]. Consequently, teachers who are also parents comprehend the significance of students’ exam scores [54]. Fourthly, teachers’ promotions and accolades are tied to students’ scores. This directly links their professional advancement to the academic success of their students, creating a strong incentive for teachers to ensure their students perform well. Additionally, the collective pride from a school’s rising status, the principal’s humanistic approach, and supportive activities (e.g., joint lesson planning and teaching competitions) further fuel teachers’ drive to fulfill the ought-to self.

Several previous studies have discussed the unclear boundary between ought-to selves and ideal selves [13,15,54]. Costa [13] stresses that participants’ ideal selves are not very different from their ought-to selves as their ideal selves might also be shaped by their duties and responsibilities. This finding is partially in agreement with the findings of this study. Most of the ideal selves possessed by participants in this study were developed around the ought-to self belonging to two categories: a teacher with good knowledge of English as an academic subject and excellent instructional practices. The two categories likely stem from the traditional culture of exam-focused English instruction in China, emphasizing language form, vocabulary, and grammar [14]. Despite this, many participants also aspired to be general educators, nurturing students’ emotional, social, and psychological well-being.

### 4.2 . Contextual influence on possible selves

As discussed in previous studies [13,15], participants’ possible selves are affected by multilayered contexts, from the immediate context (e.g., students and the curriculum) to the global context (e.g., the socio-cultural context and government policies), which is also observed in this study.

The tradition of viewing English as an academic subject in China leads to a commonality among most participants’ ideal teacher selves: proficiency in English knowledge. This contrasts with other studies [14,15,52], where ideal English teacher selves emphasize language use and communication. In China, secondary education prioritizes English written exams, resulting in a focus on grammar over communicative skills.

In addition to the global context, the formation of participants’ possible selves was also shaped by micro-level factors, including the local environment, school management, and students’ foundational knowledge and learning beliefs. As highlighted by Jack and Wynne, education management in this county has significantly improved, with policies to retain top students locally. Jack emphasized the school’s management is now more transparent and fairer. Unlike before, teachers are now recognized for their abilities, not their social background, and have more opportunities to contribute to the school’s development.

In conclusion, participants’ possible selves are shaped by various factors in both macro- and micro-contexts, with the macro-environment factors having a more notable influence.

## Conclusion and implication

This study’s key findings highlight the significant role of the ought-to self in driving motivation. Given that most related studies illuminate the central role of ideal selves and entirely ignore the role of the ought-to self, this finding will raise researchers’ awareness of the importance of the ought-to self. Teachers internalize it strongly due to their sense of responsibility and professional growth, and its importance for students’ futures. Shifts in the local context, such as reforms led by the education bureau and changes in school leadership styles, further intensify the internalization of the ought-to self, making it a more integral part of teachers’ professional roles and self-perceptions. Furthermore, the alignment of most teachers’ ideal selves with the ought-to self boosts their combined motivational power.

This study, examining seven English teachers in a Chinese high school, employs possible selves theory and an ecological perspective to probe motivation, yielding insights for related fields and stakeholders. It emphasizes two main elements of language teachers’ possible selves in China. Firstly, teachers frequently internalize the ought-to self’s expectations, considering them vital for both their students’ and their own future growth. Secondly, their ideal selves predominantly align with these ought-to self demands, boosting the motivational influence of their ideal images.

Significantly, this research not only validates the applicability of possible selves theory to studies exploring variations in teacher motivation, but also presents a comprehensive and dynamic viewpoint for understanding teachers’ motivational paths. The incorporation of an ecological perspective within the same study enriches our understanding by revealing the nuanced interaction between teachers’ inner experiences and their broader environment. It underscores how events and shifts within multi-layered contexts can significantly impact teachers’ motivation. In addition, given that most existing studies focus on English teachers in Western countries, this study contributes to this field by focusing instead on English teachers in China.

As regards practical implications, first, teacher educators should assist teachers in identifying sources of scaffolding in the external environment to achieve the requirements of the ought-to self. This could be achieved by providing teachers with training to recognize and leverage resources within their local context, such as supportive school policies, community programs, and peer networks. However, it is paramount to be mindful of the potential undue pressure that the ought-to self might impose on teachers. An overzealous pursuit of meeting the ought-to self’s requirements could inadvertently neglect students’ long-term development. Therefore, a balanced approach is essential. Additionally, the concept of possible selves should be applied to designing teacher education programs. Teacher educators can help teachers develop specific strategies to attain their ideal images, thus having a better understanding of differences between teachers. For instance, they could facilitate workshops focusing on goal-setting and strategic planning. Meanwhile, new teachers can be guided to identify the alignment between their ideal selves and the ought-to self.

While this study provides valuable insights into the motivation of English high school teachers in the Chinese context, certain limitations exist. First, due to the qualitative nature of the study and the limited participant pool, it lacks population generalization [31]. However, the study primarily aims for analytical generalization [31], achieved through an in-depth examination of participants’ experiences and their context. This approach allows readers to draw parallels and distinctions with their own experiences, thereby enhancing their understanding. Second, qualitative studies are inherently influenced by the researcher’s values and beliefs, potentially compromising objectivity. To mitigate this, the researcher involved participants in data interpretation, ensuring their insights were accurately represented. Specifically, participants were presented with summary diagrams of their data prior to the final interview, allowing for collective meaning construction between the researcher and participants, rather than a researcher-centric interpretation.

Future research is recommended to delve deeper into the role of the ought-to self in teachers’ motivation and its interaction with local and global environments. Given the significant internalization of the ought-to self observed among participants in this study, it would be worthwhile to investigate whether such a pattern persists among English teachers in China and other countries. Additionally, exploring the interplay between teachers’ ought-to selves and ideal selves could provide valuable insights. This study has highlighted the substantial impact of the ought-to self on the development of teachers’ ideal selves within a Chinese high school context. Hence, it would be beneficial for future research to examine the extent to which the development of teachers’ ideal images is influenced by ought-to selves across different global contexts.

## Supporting information

S1 Data Compressed/ZIP File Archiv. (ZIP)

## Appendix A. Teacher interview guide

1. Did you have any other work experience before becoming an English teacher in a secondary school?
2. Why did you become an English teacher in a secondary school? Did your English teachers who made a strong impression on you deeply influence your professional choice? Did your family members influence your professional choice?
3. Have you ever had any regrets about choosing a teaching career?
4. How do you see your English learning experience?
5. Do you think that teachers enjoy high social status in your environment?
6. Do you think that you suffer from a heavy workload? What are the main tasks of your job?
7. What kind of teacher did you want to be when you started teaching (ideal teacher self)? Did that change later? If so, Why?
8. What kind of teachers do you least want to be (feared teacher self)?
9. Do you think that you suffer from high pressure from your work? Why?
10. What is your typical working day like? Could you please describe it?
11. How do you think English should be taught? Has this changed a lot?
12. What do you think about educational reform such as the standard curriculum? Does this influence your practical teaching?
13.What do you think about your professional development? Do you have opportunities to have professional training?
14. What sort of rewards (if any) do you get from your current job?
15. What are you most satisfied with in your job?
16. What are you most dissatisfied with in your job?
17. Could you please describe the management in your school?
18. Do you like communicating with your students? Do you have close relationships with your students?
