# Supplementary material for: An investigation of English language teachers’ motivation from an ecological perspective: A case study from mainland China
Source: PLoS One. 2025 Apr 29;20(4):e0321139. doi: 10.1371/journal.pone.0321139 (PMC12040097; doi:10.1371/journal.pone.0321139)
Supplement: S1 Data — (ZIP) [file pone.0321139.s001.zip › data analysis results/Cali‘s summary/Cali's summary/Cali' summary 1.docx]

**Cali’s diagram 1**

But I was more diligent at that time. The boss of the institution introduced management knowledge to us, which was very helpful for me. I took notes carefully. When I had time, I practiced my pronunciation and read books. Gradually my English pronunciation was better and I felt more confident. I especially like listening to books. I can listen to a book in a few days. My knowledge was accumulated in this way.

It's not that I set a goal and worked hard to achieve it. It's just by chance. However, after I came back home and I felt it was great.

First, it is more stable in this state high school than in the private institution. Besides, it was more tired there and there was no guarantee. This thought may be affected by traditional ideas. When the private institution did not run well, I would not have a job any more.

I did not prepare much for the exam. It may because that I was lucky, or fewer people participated in the exam. Finally, I passed the exam. Since it was not easy to pass the exam and I heard that high school teachers should have a postgraduate degree last year, I decided to come back. I resigned and came back.

The previous private tutoring institution was very helpful to me in case of letting me know some effective methods to memorize words.

I didn't want to come back to my hometown. My mom really wanted me to come back. My friend also suggested that I could take the examination for recruiting teachers of state schools.

My mother told me that if I could pass the exam, I should come back. However, if I failed, she would not force me to come back then.

I gained confidence through my teaching English and making individual small progress. In addition, I accumulated knowledge related to English. Moreover, I improved my management ability.

In daily life, I am not good at communicating with adults, but I was more confident in communicating with students in class. As a teacher, I need to communicate with students and their parents. I could communicate with them more efficiently than I did with others in my daily life. I liked doing concrete things and solving problems one by one by. I mean when I found children’s problems, I would consider how to help them solve these problems.

I also felt happy when I was important to others.

At the beginning, I didn't think of being a teacher, but later wanted to make some money by teaching English. However, by doing part-time jobs, I found that I liked being a teacher and I was good at it. I felt happy to be with children as they had high emotional intelligent. On the one hand, when I could help them, I felt happy. On the other hand, being a teacher enabled me to feel a sense of fulfillment.

Since I became an English teacher, my personal characteristics changed a little bit and I became more confident.

Passing the exam for recruiting high school teachers in a public school

I was so scared at the beginning, but I became more confident at the end. Finally, I was the leader of the English group. In the past one and a half years there, I had grown up much more than I did in the university. It was just like another university of mine. I think that such experiences helped me quickly adapt to the high school teaching here.

Influence from others

Bein**g** an English teacher in a private language training institution

Being a part-time English teacher
