# Supplementary material for: An investigation of English language teachers’ motivation from an ecological perspective: A case study from mainland China
Source: PLoS One. 2025 Apr 29;20(4):e0321139. doi: 10.1371/journal.pone.0321139 (PMC12040097; doi:10.1371/journal.pone.0321139)
Supplement: S1 Data — (ZIP) [file pone.0321139.s001.zip › data analysis results/Cali‘s summary/Cali's summary/Cali' summary 2.docx]

**Cali’s diagram 2**

I think it was the God who helped me make choices all the time.

When I Watched TV series, I thought foreign languages were attractive. In this case, I learned business English as my major purely by accident.

The educational level of my parents are low and my mother didn't graduate from the primary school. In this case, they didn't know how to choose a major for me. I chose majors based on my superficial understanding of their name. The name “Business English” was attractive to me.

However, when I began to study as a student of this major, I felt great. I like some part of the alien culture. I also liked to watch inspirational movies in English, especially those movies exploring simple and pure things, such as human nature. Thanks to my English learning, I am more confident. I know that I can be different and have many possibilities. I even could not remember my choice of major clearly. My choice of this major was accidental and I am lucky that I made the right choice.

I think it was relatively successful but inefficient. Now my learning ability has been improved a lot. In fact, it is critical to find a right method, like doing a lot of other things. I started to learn English in grade one in my middle school. It was very hard for me to learn English. Therefore, I had to mark them with Chinese. At that time, the English teacher was very strict, and I was also the representative of the English subject. The teacher required us to memorize every English text, and a lot of hard work had to be done.

After watching them, I was often touched. There is a movie called The Weak Side, which talks about the influence of parents’ words, though and deeds on children, the topic of which was rarely found in domestic movies. The movie was about a vagrant black boy, who was tall and fat, and discriminated against by other people. But a woman provided him clothes and all kinds of help for him. We all need to learn from this lady. This kind of movie explores the kindness of human beings and this should be passed on. These movies lead us to discover the good in people’s nature.

Others like the Pursuit of Happiness, Forrest Gump. Those movies really touched me. They really inspired me and liked those movies.

If I had majored in other majors, I might watch movies related to my major to improve myself. But I learned English, and it's related to education. Therefore, I wanted to watch movies related to education methods and communication with people.

At the beginning of learning, teachers suggested us to listen to English songs and watch English movies. At the beginning of watching movies, I paid attention to the subtitles to see their meaning and understandings of its ideological content were shallow. I was more concerned with the pronunciation and spelling of words, and the meaning of sentences. However, after further learning English, I began to pay attention to the characters' language, expressions and actions. I had a sense of substitution. I felt that the expressions and words of the protagonist expressed the ideas I wanted to express. At the beginning, I did not understand the movies very thoroughly. The black humor in it may not be understood, but after learning more, I gradually understood the humor in it.

Learning experience

Choosing to major in business English at the University University
