# Supplementary material for: An investigation of English language teachers’ motivation from an ecological perspective: A case study from mainland China
Source: PLoS One. 2025 Apr 29;20(4):e0321139. doi: 10.1371/journal.pone.0321139 (PMC12040097; doi:10.1371/journal.pone.0321139)
Supplement: S1 Data — (ZIP) [file pone.0321139.s001.zip › data analysis results/Cali‘s summary/Cali's summary/Cali' summary3.docx]

**Cali’s diagram 3**

I'm a bit of learning by doing. In daily life, I am not good at communicating with adults, but I was more confident in communicating with students in class. As a teacher, I need to communicate with students and their parents. I could communicate with them more efficiently than I did with others in my daily life.

I liked doing concrete things and solving problems one by one by.

I was not good at talking, introverted, shy and not very good at talking. After learning English, I was a little more outgoing.

My personal characteristics changed a little bit and I became more confident

When I was in college, I was a person with a low sense of presence. My Pronunciation of Putonghua and English were not standard. When I had to make a speech, I felt awkward. I dared not speak when teachers asked us to introduce ourselves as I lacked confidence. When I first joined the private institution, I was the same as when I was at my university. But I was more diligent at that time.

Her personality

The first stage goal was achieved while the second goal has not been achieved. The rank of students’ scores has something to do with the size of the class. In addition, there is a student with autism in our class who lags behind in academic performance. Moreover, there is also an art student with poor academic performance. Because of these two students, the average score of my class is lower. I hope that my second goal can be realized by the end of this semester.

I don’t want to be a teacher who only impart knowledge without cultivating students’ mind (without close connection with them).

I feel that no matter whether students learn English well or not, at least, I should let students know that they should work hard. I hope my values can be recognized by my students. I want my students feel my love and they can be responsible for themselves with their work hard.

In the daily meeting for teaching research, the knowledge points explained carefully by veteran teachers seemed to shine, and I was really attracted by the charm of knowledge.

I was happy in the process of lesson preparation. Doing things carefully which can improve students had a positive impact on the teaching motivation.

At that moment, I found that I was so fond of my job. Because of this job, I can work together with those young people towards one goal. Everyone needs to grow up.

First, I want to help my students outperform their counterparts in another group (class 15 /class 37) (class 1- class 21, class 22- class 42). Then I want my two classes of students to be ranked at the top two among B-level students.

Attitudes towards the job
