# Supplementary material for: An investigation of English language teachers’ motivation from an ecological perspective: A case study from mainland China
Source: PLoS One. 2025 Apr 29;20(4):e0321139. doi: 10.1371/journal.pone.0321139 (PMC12040097; doi:10.1371/journal.pone.0321139)
Supplement: S1 Data — (ZIP) [file pone.0321139.s001.zip › data analysis results/Cali‘s summary/Cali's summary/Cali' summary4.docx]

**Cali’s diagram 4**

I prefer to be an expert teacher……. Expert teachers are excellent teachers who have advanced educational ideas, a high command of English subject knowledge and teaching skills.

If I can go abroad to study for two years, my oral English will be improved. Then, students may admire my oral expression and I am more persuasive as an English teacher. I can give students more in-depth explanations and explain problems from a brand-new perspective.

I particularly admire people who can pronounce English like natives. Students also admire those charming teachers who pronounce English beautifully

I think that education is a process in which a person use language to ignite another person’s enthusiasm for improvement. Therefore, the ability of expression is very important. I want to improve my communication ability and to communicate with students more efficiently. Moreover, I am more willing to speak to others with confidence.

I feel that no matter whether students learn English well or not, at least, I should let students know that they should work hard. I hope my values can be recognized by my students. I want my students feel my love and they can be responsible for themselves with their work hard. In my opinion, students can be attracted by my personal characteristics in this way, and students will make progress in English according to the plan made by me. I believe that the process of education is that educators ignite others’ aspiration for becoming better.

At this moment I realized the mission of being an educator, which is to awake a child’s inner spirit, which is full of love, positive energy and beauty.

I will put the interesting things into my class. In addition, I use some popular words when I teach. Maybe they think I'm energetic.

In another sentence, when students set a goal and fail to achieve it, they should consider the failure is caused by either they did not work hard or the wrong methods. They need to try other methods, if it still doesn't work, they need to ask help from others, such as their peer students and teachers.

I want my students to learn that they should try to solve problems rather than let themselves indulge in negative emotions.

I tell my students that you don't need to do so many exercises, but you have to think deeply and flexibly.

.

Other teachers praised my students for their thorough explanation for exercise questions with concise language in one of my demonstration classes. I think the effect was also good. I think this kind of training can help students accumulated experiences.

But my students perform better because I would let them do something by themselves. I like a director to give then some guidance when they have problems.

My goal was to get students involved in the class. ….There were problems with their pronunciation and sentences when they performed the drama. However, the general meaning of the drama could be understood by audiences. It can be seen that students were devoted and tried their best. I think these activities benefited students and they could have some special feelings for English.

Yeah, there are some advantages, such as I was able to engage students in class without cramming them all the time.

As a teacher, I think as long as I can improve my students, I have achieved my goal I don't care what level of students assigned by the school to me. If leaders want me to teach A class, I will try to do it. I am also adapted to B level students.

Teaching beliefs and methods
