# Supplementary material for: An investigation of English language teachers’ motivation from an ecological perspective: A case study from mainland China
Source: PLoS One. 2025 Apr 29;20(4):e0321139. doi: 10.1371/journal.pone.0321139 (PMC12040097; doi:10.1371/journal.pone.0321139)
Supplement: S1 Data — (ZIP) [file pone.0321139.s001.zip › data analysis results/Cali‘s summary/Cali's summary/Cali' summary5.docx]

**Cali’s diagram 5**

I think that teachers can enhance their teaching motivation when they take the examination together with students as this helps teachers reflect on their shortcomings. At the same time, they can think in the students' shoes, which is helpful to their teaching career.

The environment of our school is changing now, and the competition among teachers is fierce. Now there's a word called Neijuan. During my fist year here, I had some free time. But now I feel tired. From the second year, there has been a new headmaster. I think these changes are connected with the current headmaster's management style, which really stimulates everyone's enthusiasm.

There will be a monthly examination for our students. Because there is a ranking for students’ grades of each examination, I have pressures.

The students' growth makes me have a sense of accomplishment

In addition, some of my colleagues now are my teachers when I studied here. I also learned a lot from them in respect of teaching skills and knowledge.

I knew that the teacher had spent a year in Britain. She was so confident. I was deeply shocked by her and I was also aware that there has been a significant space for me to improve myself. I should practice more my oral English and the intonation of it.

Relations between our colleagues are quite harmonious.

Many of the teachers who teach us now had stayed abroad, and they inspired us a lot.

The teacher had a sweet voice and super affinity, and was very good at interacting with classmates. Her way of communication with students was particularly worthy of my learning. I was greatly motivated by wanting to be as good as my teacher.

Yes, they support me. They all think this is a sacred profession. A teacher’s several words may have a big impact on students. They always ask me whether I prepare well for my class. As long as it is related to the work, they support me.

Support from family member

I get along well with students as friends from the very beginning. We are relatively harmonious.

Most of students gave me high scores for the evaluations and some of them even evaluated me as A+++. I was quite happy

Their enthusiasm motivated me a lot. The representative student worked carefully and listed out students’ questions, such as some grammatical rules. This kind of considerate behavior also has a positive impact on the teaching motivation.

When students were in a state of accepting knowledge, both the classroom atmosphere and teaching efficiency were greatly improved, which encouraged teacher’s morale and formed a virtuous circle.

Women teachers, in particular, have no chance to go out after having children….. For example, I have a colleague whose wife is also an English teacher. His wife assumes a lot of family responsibilities.

The influence of postgraduate study

Students’ influence
