# Supplementary material for: An investigation of English language teachers’ motivation from an ecological perspective: A case study from mainland China
Source: PLoS One. 2025 Apr 29;20(4):e0321139. doi: 10.1371/journal.pone.0321139 (PMC12040097; doi:10.1371/journal.pone.0321139)
Supplement: S1 Data — (ZIP) [file pone.0321139.s001.zip › data analysis results/Cali‘s summary/the list of Cali.docx]

**The codes list of Cali**

| The overarching codes for the interviews | The overarching codes for the reflective journals |
| --- | --- |
| the management of the school | the management of the school |
| teaching belief and methods | teaching belief and methods |
| support from family members |  |
| students' influence | students' influence |
| reasons for being a state high school English teacher |  |
| reasons for being an English teacher |  |
| ought to teacher self |  |
| the influence of postgraduate study | influence of current postgraduate study |
| the influence of colleagues | the influence of colleagues |
| ideal teacher self |  |
| feared teacher self |  |
| being an educator | being an educator |
| being a female teacher |  |
| attitudes towards the job | attitudes towards the job |
