# Supplementary material for: An investigation of English language teachers’ motivation from an ecological perspective: A case study from mainland China
Source: PLoS One. 2025 Apr 29;20(4):e0321139. doi: 10.1371/journal.pone.0321139 (PMC12040097; doi:10.1371/journal.pone.0321139)
Supplement: S1 Data — (ZIP) [file pone.0321139.s001.zip › data analysis results/Harley's summary/Harley' summary 5.docx]

**Harley’s diagram 5**

But today's students have strong personalities. Moreover, my time is limited. If some students’ scores are not good, I will talk to the students. But it is not effective.

I have very short time for having a rest. I am always very busy the whole day. I hardly have the weekend. I need a lot of time to prepare lessons, and it also takes a lot of time to comment students’ homework. Especially, it takes a long time to read students’ composition. Some teachers finish all these tasks at school. But I need to being my tasks to my home to finish them. After my children fall asleep, I then continue to prepare lessons.

The requirement of the school was higher and more strict year after year. The school requires teachers to arrange a test for students every week. In addition, there is a homework task on every Wednesday. Students' homework needs to be checked in time and give them feedback timely.

The management of the school

At the beginning, there was not much difference in age between me and my students, and I had a good relationship with them. I had plenty of time to spend with my students. Now, a lot of students still keep in touch with me.

When they had questions, they were willing to ask me for advice, including questions in their private life. I was like a sister of them.

I never yell at students. I am very patient to help students solve their psychological problems. I am the one who intend to praise students exaggeratedly.

My tutor was very professional with a low profile. I was very lucky to have an opportunity to learn from my tutor and he gave me many useful suggestions.

One of my colleagues encouraged me to take part in it.

On Wednesday, teachers in our group asked me to demonstrate again. Our colleagues were very kind and gave me a lot of suggestions, and improvement measures. I thought about what they said carefully.

Some teachers in our group praised me highly of my courseware and course design. A colleague left me a note saying that I had gone beyond myself as she knew more about my personality. Therefore, I appreciate the affirmation about me from my colleagues very much.

I can't tell my colleagues these family problems, and I can't look sad at work. Therefore, I have to internalize these negative emotions.

I have a colleague. She is very energetic and passionate. She was able to motivate students no matter what kind of state students are in and feedbacks given by students.

I can realize it when other teachers give me advice. As my colleague said in the message to me, I was beyond myself. I was trying to break through myself and overcame my character defects.

When I was not married, I liked to tell my mother a lot about myself. But now my mother is busy taking care of her grandchildren. She said I should not work so hard, and I need to take good care of my health. My mother didn't approve of my efforts, so I stopped telling her. My husband gets annoyed if I talk to him too much.

My husband suggested me that I could go back to primary schools and I would not so busy.

My husband wants me to spend more time with the kids, and complained that I spend too much time at work. He does not understand me.

Influence of family member

Influence of colleagues

Influence of students
