# Supplementary material for: An investigation of English language teachers’ motivation from an ecological perspective: A case study from mainland China
Source: PLoS One. 2025 Apr 29;20(4):e0321139. doi: 10.1371/journal.pone.0321139 (PMC12040097; doi:10.1371/journal.pone.0321139)
Supplement: S1 Data — (ZIP) [file pone.0321139.s001.zip › data analysis results/Harley's summary/Harley' summary1.docx]

**Harley’s diagram 1**

And I always wanted to be a translator. So I went to a chemical factory to be a translator. After being a translator, I still wanted to be a teacher or from my heart.

I am a very down-to-earth person, kind of silly. The night shift really influenced my health negatively. Compared with being a teacher, being a translator does not have regular time to rest.

Being a translator was my ideal but it cannot bring me a stable life.

Being a translator

English learning experience

I was not good at Chinese and math. I can complete seriously the homework assigned by my English teacher. Gradually, the more I learn English, the more I was interested in it. I became more and more interested in learning English as I can learn it. I can understand what the teacher said, and I can accept it faster than other students. Also I was willing to learn it as I had a sense of accomplishment when I learn English.

Moreover, but my parents didn't agree that I went back to the school in the village to be a teacher. Conditions of the rural school was extremely difficult.

My parents thought that I was not suitable to be a teacher as I always lost my voice. It may because that my throat did not cover completely. Therefore, they suggested me to give up.

But I did not want to give up them. I thought that they were eager to learn well. I said to them that when I extended my hand to you, you should also extend your hand to me. I want to be a teacher who can help students solving their learning difficulties. I do not want to give up any student.

Tag：Is it a rural primary school?

Harley：A middle school.

Tag: The conditions were tough, right?

Harley：Yes, extremely.

I gradually found myself liking and enjoying teaching. Being a teacher is actually a process of energy transfer. I can influence students and lead them to become more excellent while accompanying them to grow up.

The experience of being an intern teacher influenced me significantly.

I was willing to. Because I worked so hard at that time. I volunteered to tutor students, and I worked late. At that time, I lived with students together. I went back to my dormitory late at night. I was very hungry and I had to cook by myself then. At that time, my health was not good.

They wanted me to have a stable position by passing the exam for recruiting teachers. They thought that it was stable for a girl.

I had been taken care of by my parents well and I followed their advice and returned to my hometown.

The influence of parents

The performance of the university entrance exam

I didn't do too well in the university entrance exam. Therefore, I apply for a school near my hometown.

I love being a teacher. This has no relation with money and being successful in career. I enjoy the feeling that when I stand on the platform, I can transmit what I have known to other people. The sense of achievement can not be described by words when I see that students nod their head to show their understandings.

Being an intern English teacher in a rural middle school
