# Supplementary material for: An investigation of English language teachers’ motivation from an ecological perspective: A case study from mainland China
Source: PLoS One. 2025 Apr 29;20(4):e0321139. doi: 10.1371/journal.pone.0321139 (PMC12040097; doi:10.1371/journal.pone.0321139)
Supplement: S1 Data — (ZIP) [file pone.0321139.s001.zip › data analysis results/Harley's summary/Harley' summary2.docx]

**Harley’s diagram 2**

If I'm not well prepared, I'm not confident. My personality has been like this since I was a child, and I always want to change. My husband often asks me why I am not confident. I am no worse than others. However, I tend to see my own shortcomings and magnify the advantages of others.

I was an introverted person. In high school, I did not communicate with others actively. but I was eager to talk with others if they can initiate the talk.

I am very sensitive to and care about other people's opinions.

I was introverted and didn't communicate with others very much. This praise made me feel good. My parents did not praise me much. They just thought that I was very obedient, but they never praised me in front of others. I desired to be recognized and praised.

I am more introverted, since childhood. I am not open. I am not particularly energetic and passionate.

I hoped their grades can be improved as much as possible.

I was very strict with them, and their progress was obvious. They had a solid foundation, and their grades were good, ranking the first and second.

In addition to teaching and checking homework, I also hope to help students after class. I want their grade for the university entrance exam can be higher and the grade of their English should not lag behind.

The ought to teacher self

In addition to my routine teaching, I need to take care of the children. I feel very helpless. I also want to have time to read. But I can not ignore the child. I am now in the state of falling to take good care of child and work well.

I have two kids now, and the quality of my sleeping at nights is poor. I don't have anyone who can help me take care of my kids. My husband is too busy to do it. I am tired.

Later, I got married and I did not have enough time and energy to work well. My work state changed.

I now have two children, and they are relatively little. I did not have enough energy.

My biggest concern is that my children are small and taking care of them occupies much of my energy.

For the first three years of my career, I spent almost all of my time in school. After class, I would find students and tutored them privately. Sometimes I taught them from the very basic knowledge. It may even that of middle school.

I often stay up late to get things done. I can actively complete the tasks required by the school

Her personality

Attitudes toward the job

Being a female teacher
