# Supplementary material for: An investigation of English language teachers’ motivation from an ecological perspective: A case study from mainland China
Source: PLoS One. 2025 Apr 29;20(4):e0321139. doi: 10.1371/journal.pone.0321139 (PMC12040097; doi:10.1371/journal.pone.0321139)
Supplement: S1 Data — (ZIP) [file pone.0321139.s001.zip › data analysis results/Harley's summary/Harley' summary3.docx]

**Harley’s diagram 3**

I want my routine reading class teaching can be similar to the demonstration class in terms of my in-depth understanding about the content of the text.

I want to teach in English and improve my courseware design.

I need to improve my grasp of the text to have a crystal idea of what should be mastered by students, what kind of ability of them can be improved, and what kind of humanistic knowledge should they understand.

I want to teach in English and enable the students to participate with English.

To sum up, I lack of confidence, expression management and the ability to mobilization of students' enthusiasm.

I have a lot of skills in doing exercises, and I want to summarize them, but I do have systematic time.

My dream is to influence more people. I think the profession of teacher is to influence a lot of people with his own ability. His words and his way of life will make many students change, and may even affect their whole life.

I just exert great effort, wanting to help the students become better and more excellent. Then they can understand my instruction and learn English well. That's what I try to tell my students.

I hope to understand each student's learning difficulties, and help students to solve their difficulties. Therefore, students have a significant improvement. I want to help them with my best

I hope that there was no distance between me and the students.

My students got good grades and I won honors from the school. Later, I got married and I did not have enough time and energy to work well. My work state changed.

My time and energy are limited right now. My relationship with my students is not that close anymore. I am unable to check my students' homework as frequently as before.

To sum up, I lack of confidence, expression management and the ability to mobilization of students' enthusiasm.

Although I am not lively, and not cheerful, they like me.

I always bring my negative emotions to my home. I'm not very good at balancing taking care of family and work.

The leader of our grade appreciated my ability as my students had good grades. I seldomly asked for leave and I did not have many things to distract me from my work.

I was confident in my professional knowledge. I like English very much. In addition, I majored in English in university. In terms of teaching skills,

I don’t want to be a teacher who cannot let students understand his or her instruction. I gave up physics in the university entrance examination. The reason for I giving up was that when I summoned up the courage to ask the physics teacher a question, the teacher did not explain it clearly to me. In fact, I am very talented in physics. But because the teacher himself is a little confused about the topic, I began to reject physics. Later, I thought that if the teacher at that time had enough professional knowledge to let me understand the topic, maybe I would not give up physics. The score of my university entrance examination would be better.

The second kind of teacher I don't want to be is a teacher who hurts students' self-esteem. My head teacher in high school always liked to twitch his lips. I thought that he was looking down on me. I later learned that it was a habit of his. I was an introvert. I took it personally and it hurt my self-esteem. Some students may perform poorly in study, but they also have their own shining points. For example, he is polite, and he has various possibilities in the future.

Ideal teacher selves

Current teacher self

Feared teacher selves
