# Supplementary material for: An investigation of English language teachers’ motivation from an ecological perspective: A case study from mainland China
Source: PLoS One. 2025 Apr 29;20(4):e0321139. doi: 10.1371/journal.pone.0321139 (PMC12040097; doi:10.1371/journal.pone.0321139)
Supplement: S1 Data — (ZIP) [file pone.0321139.s001.zip › data analysis results/Harley's summary/Harley' summary4.docx]

**Harley’s diagram 4**

Then I found that I improved myself obviously and had a crystal understanding about the key and difficult knowledge points. Therefore, I can grasp the key points in my teaching. I can then decide whether the words should be expanded, or not. I mean that students only to know these words when they encountered them. There was no need for much expansion of these words.

I was very strict with them, and their progress was obvious. They had a solid foundation, and their grades were good, ranking the first and second. The main reason was that I spent a lot of time to help students overcome the difficulties in their learning.

One is basic knowledge, and the other is their psychological state. ……I encourage them to let them realize that I'm teaching from the basics

his time I did not enter the next round, because I failed to mobilize the enthusiasm of students and enabled students to actively participate in the class. But my demonstration class is similar to my routine ones. I did not ask students to preview in advance.

It turns out that I can still find the time to do something. In this competition, I have improved a lot. I also learned to make a video, and prepared a courseware carefully. I prepared to teach in English. I improved myself in terms of controlling class time, and deeply decoded the text.

Therefore, although I prepared the courseware well for the intra-group competition, my control of time and procedure were not ideal as there was no time for me to do the lecture trial. I taught new content, and the students did not preview. I thought that it was not good to teach the content the students have learned. Hence the students did not cooperate well. Because of my insufficient preparation and lack of confidence, I did not perform well. I barely passed the group competition. For the next round, I had enough time for preparation.

My biggest concern is that my children are small and taking care of them occupies much of my energy. In addition, I am teaching students of B-level and I do not use English much in my teaching.

First, there is the inter-group teaching contest which means that there are two teachers from each subject within the group. Second, there is the teaching contest between teachers from different subjects within the group. Then, there is the teaching contest for this semester. Finally, there is teaching contest for the year. This is the second year for this teaching contest. Last year, because of the pandemic, there was not the final teaching contest.

I think of myself as a student. From the perspective of a student, I consider which parts of the knowledge points students may have problems with. I did not intend to give students a lot of expanded and difficult knowledge points. I would like them to master the basic ones. Some teachers like to expand rich for new words. But I like to lay a solid foundation for students. Therefore, I just gave them a thorough explanation for the key usage and collocation of new words. In another sentence, I was from the students’ perspective to pay attention to the most simple and basic knowledge points. I was the kind of teacher who liked to lay a solid foundation for students

Participating in the teaching contest

Teaching beliefs and methods
