# Supplementary material for: An investigation of English language teachers’ motivation from an ecological perspective: A case study from mainland China
Source: PLoS One. 2025 Apr 29;20(4):e0321139. doi: 10.1371/journal.pone.0321139 (PMC12040097; doi:10.1371/journal.pone.0321139)
Supplement: S1 Data — (ZIP) [file pone.0321139.s001.zip › data analysis results/Jack's summary/Jack's summary1.docx]

**Jack’s diagram 1**

I am interested in learning languages.

In summary, I had some interests in learning English. When I found that I was able to learn English well, my interest became stronger.

When I transferred to another school and met this new teacher, she taught from the phonetic symbols. It seemed to be a backward method now. At that time, as soon as I learned the phonetic symbols, I could read English words. Then I felt the great sense of accomplishment. This thing opened the door for me to learn English. Then I think it is easy to learn English.

Yes, when I graduated in 2006, jobs were already hard to find.

English learning experience

As my family was poor, I did not want my family to support me anymore. Therefore, I wanted to find a stable job to support myself as soon as possible.

Family background

When I was young, I wanted to go outside, and I thought it was boring to be a teacher

Unwilling to be a teacher initially

Difficult to find a stable job

My parents don't have much knowledge. My mother left school after her fourth grade in primary school and my father had a low education level. They wanted me to have a stable job and an income.

The influence of family member

The choice of English major

Yes, I learned English literature. I chose this major because graduates of this major could find a job more easily. When I chose my major, the head teacher told us that graduates of English major would find jobs more easily in the future.
