# Supplementary material for: An investigation of English language teachers’ motivation from an ecological perspective: A case study from mainland China
Source: PLoS One. 2025 Apr 29;20(4):e0321139. doi: 10.1371/journal.pone.0321139 (PMC12040097; doi:10.1371/journal.pone.0321139)
Supplement: S1 Data — (ZIP) [file pone.0321139.s001.zip › data analysis results/Jack's summary/Jack's summary2.docx]

**Jack’s diagram 2**

I began to pay attention to the promotion of professional title as some of my excellent colleagues already participated in the evaluation process. Moreover, this promotion means that my salary can be increased.

And in recent years, to be a teacher with senior professional titles is the biggest motivation for my teaching. There is more quota for teachers to be promoted with junior professional title than senior professional titles.

I like being a teacher. In the school, it is very objective as knowledge is admired. In addition, there are students, who are very young and lively. Staying with them makes me feel myself young. I also feel a sense of accomplishment. I can rely on students to achieve the goals I didn't achieve when I was young. They can do better than me.

professional title promotion

If the teacher is not qualified, there will be some punishments. For example, the teacher may be asked to teach students with lower grades or poor academic performance. It may also be that teachers who are not qualified are banned to teach for a period of time. I was affected by those methods. If the average grade of students of a class ranks from the bottom or students’ evaluation scores of a teacher ranks from the bottom, the teacher is not allowed to teach students of grade three.

There was a time when one of my colleagues was banned from teaching because the students didn't think highly of him.

I used to be in a hurry and imitated others’ methods mechanically. But I encountered problems in the process. I then understand that teachers’ personal characteristics influence a lot. I am different from those teachers who are gentle and flexible.

People are competitive and want to make themselves better. In this way, they can grow up step by step.

Personality

The main stress is from the university entrance examination. If the test scores of my students are not good, their parents will be very disappointed. The school also evaluate teachers in terms of students’ scores.

This is directly related to the global goal of the school, which is to help more students to be enrolled by the top universities, such as the Tsinghua and the Peking university.

Teachers must put students' scores in the first place. Grades are important for students and their parents and families.

Attitudes towards the job
