# Supplementary material for: An investigation of English language teachers’ motivation from an ecological perspective: A case study from mainland China
Source: PLoS One. 2025 Apr 29;20(4):e0321139. doi: 10.1371/journal.pone.0321139 (PMC12040097; doi:10.1371/journal.pone.0321139)
Supplement: S1 Data — (ZIP) [file pone.0321139.s001.zip › data analysis results/Jack's summary/Jack's summary3.docx]

**Jack’s diagram 3**

A good teacher is not only good at teaching but also managing students. Sometimes management is even more important than lecturing.

Therefore, I want to learn from successful experience from other subjects and improve myself gradually. Hence, I can help others, leading teachers of my group to conduct teaching and researching activities more vigorously. This is a strong motivator for me.

Knowledge transmission is in the first place as the university entrance examination is the most critical.

The traditional cramming method is not accepted by the students. If students admire teachers’ knowledge and ability and teachers can find the right way for students to acquire knowledge, there will be quite efficient. The teaching methods need to be flexible and the classes need to be interesting. As students also have a wider range of knowledge than before, the requirements for lesson preparation are higher than before.

Being a leader of the joint lesson planning team

For example, we used to work alone and prepare our own lessons. Now, with the development of English textbooks, the change of students and the development of the Internet, students have higher and higher requirements. The traditional cramming teaching mode cannot satisfy the students now. Additionally, the university entrance examination tends to assess students’ ability. Therefore, the previous simple and mechanical way to help students memorize knowledge is not effective. Both students and teachers are also very tired.

I also realized the importance of the teaching research activity. Only by high quality teaching research activities, can we find ways to break the bottleneck.

Today, the English group started to prepare the "English Subject Teaching and Research Workshop". The school required us to gather the achievements of excellent teachers and show them to everyone. This is a very good opportunity to make a good start for the English group's teaching and research activities.

I felt the pressure as the group leader of lesson planning. I determine to move forward the teaching research activity, improve the quality of joint lesson planning and students’ management.

Teachers need to have excellent professional ability.

Teaching belief and methods
