# Supplementary material for: An investigation of English language teachers’ motivation from an ecological perspective: A case study from mainland China
Source: PLoS One. 2025 Apr 29;20(4):e0321139. doi: 10.1371/journal.pone.0321139 (PMC12040097; doi:10.1371/journal.pone.0321139)
Supplement: S1 Data — (ZIP) [file pone.0321139.s001.zip › data analysis results/Jack's summary/Jack's summary4.docx]

**Jack’s diagram 4**

The excellent professional ability means that teachers have rich knowledge, and can explain the knowledge thoroughly, so that students can understand it clearly.

I want to cultivate students who really love English learning and their achievement in English is greater many times than me. After I get my highest professional title, I want to active the classroom atmosphere genuinely. Then I want to focus on cultivating students' interest. Now it is a matter of finding a balance between ensuring students’ high grades and fostering their interest.

After I can answer students’ questions, I wanted to make the class more interesting and active and helped students become more efficient in their learning.

I found some materials and videos. It was easy to activate class atmosphere as there was lots of multimedia materials. I added rich and vivid examples besides my explanations.

Being a qualified teacher and answering students’ questions are the initial requirements. These are the basics.

I wanted to be a qualified teacher who can stand on the podium with sufficient knowledge (zhanwen jiangtai).

Because of that teacher, I changed my overall view of English. Then I thought that English learning was very easy.

I began to focus on students' learning efficiency. It depends on what level of students the teacher is teaching.

Student grades account for 60 percent. Others include inspection of teachers’ lesson plans and some other routine inspections and evaluations.

Yes, especially I have been evaluated as a backbone teacher (gugan jiaoshi) for three consecutive years.

Since 2012, I became a headteacher, which was a huge turning point in my work. After serving as the headteacher, I came into contact with a higher platform and had a deeper understanding of the students. I also had one more assessment, which was the management of the class.

Yes, but this goal (being a backbone headteacher) is relatively small and usually easy to achieve.

Three years ago, when I started to be the group leader, I always felt that my own way was right and I didn't accept others' opinions. Later, I found that we were very tired under my leadership, but there was no achievements.

Yeah, according to the requirements of the school, a teacher who has been evaluated as a backbone teacher (gugan jiaoshi) for five consecutive years can become an eminent teacher.

I don't think I realize the goal of delivering high efficient lessons. I have had this goal for a long time. I want to pay more attention to the actual effect of the lesson.

I felt the pressure as the group leader of lesson planning. I determine to move forward the teaching research activity, improve the quality of joint lesson planning and students’ management.

Being a backbone teacher

Being a leader of the joint lesson planning

Ideal teacher selves
