# Supplementary material for: An investigation of English language teachers’ motivation from an ecological perspective: A case study from mainland China
Source: PLoS One. 2025 Apr 29;20(4):e0321139. doi: 10.1371/journal.pone.0321139 (PMC12040097; doi:10.1371/journal.pone.0321139)
Supplement: S1 Data — (ZIP) [file pone.0321139.s001.zip › data analysis results/Jack's summary/Jack's summary5.docx]

**Jack’s diagram 3**

I began to teach high quality students. The rough and easy way didn't work. When I was strict, the less likely it is to work. Students’ ideas have changed. Teachers need to have excellent professional ability. Moreover, they need to face students with genuine love. The teacher needs to discuss the solution to the problem with students.

I want to teach good students and grow quickly.

I was confused and even annoyed with the students. I was young at the time and felt that the students were attacking me personally. Later, I realized I needed to work hard as at that time most students’ family were poor and they did not have much money.

When I have a good relationship with students, the students like me. I pay attention to student's personal difficulties and help them consciously. When I saw a student feel down, I gave him or her encouragements. I also comforted them if they were criticized by other teachers. Therefore, I can establish a closer relationship with my students.

They had limited money for meals and some of them ate steamed bread and spicy slices. Their lives were not easy and I was touched by that. I still had to prepare my courses until twelve o 'clock at night.

My wife, who is also a teacher, is very supportive and understanding of my work.

The rise of my salary in recent years has been quite remarkable. I don't ask much of life. At least now my income matches my labor.

The global environment has changed. The whole management for education becomes more mature and formal. At the time of my initial entry, the requirements were not strict. Later, there have been identified assessment items, and higher requirements for teachers. I improve myself gradually.

The development of our school is based on a quantitative system of evaluation. The previous principal introduced the quantitative system of evaluation. The current principal has further developed the quantitative system of evaluation. In addition, he has high emotional intelligence and can understand what the teachers need. He uses effective incentives to motivate teachers.

Students’ influence

My growth is inseparable from the tradition of the school and the power of fine examples of these experienced teachers. Whenever I am confused, they are guiding me step by step. Every time I encounter any difficulties, there is always a light, which can guide me to seek help. Therefore, I gradually grow up to be myself now.

Students’ academic performance is fundamental to the development of the school.

At that time, teachers in our English group were excellent, and they were my models. I was not an excellent person but by following steps of my model, I grew up gradually.

My colleague was ranked the first for the teachers’ evaluation by students. This was really touched me as he was ranked the last previously.

Experienced teachers were tired of my questions as I asked them questions every day. There were a dozen of new teachers like me, who liked to ask experienced teachers questions.

Support from family member
