# Supplementary material for: An investigation of English language teachers’ motivation from an ecological perspective: A case study from mainland China
Source: PLoS One. 2025 Apr 29;20(4):e0321139. doi: 10.1371/journal.pone.0321139 (PMC12040097; doi:10.1371/journal.pone.0321139)
Supplement: S1 Data — (ZIP) [file pone.0321139.s001.zip › data analysis results/Lily's summary/LIly' summary5.docx]

**Lily’s diagram 5**

I am a straight shooter and sometimes I make my colleagues unhappy. But basically I have a good relationship with my colleagues. For young teachers, I will give them some advice based on my own experience.

spected teacher with rich teaching experience in our group talked about his ideas. I thought that the teacher did excellently.

As there was an increase in the difficulty of knowledge, I observed other colleagues’ lessons, read the textbooks and auxiliary material carefully.

I realized my disadvantages. A few days ago, a respected teacher with rich teaching experience in our group talked about his ideas. I thought that the teacher did excellently.

The grades of my class are very good, but my students did not get the honor of excellent class and I did not get the honor of excellent head teacher. I felt sad.

I want to learn from teachers who are down-to-earth. I don’t work meticulously and conscientiously as they did.

After all, students are human beings with souls and flesh, and they have the right to choose for themselves.

At present, I am more tolerant to students with various characteristics. I think this is what the young people should be like. if they are the same as our ideal image. The world is not wonderful. Everyone should have their own characteristics

I am so happy that students of one of the classes I am teaching improved their grades significantly. The average grade of them placed the third. It was effective that I help them pay more attention to basic knowledge and writing.

As a mother of two children, I feel sorry for my kids, and I want to spend more time with them.

However, my grades were not high and I was at the age for marriage. I had to stay there to teach.

There was a period of time during which my only work is teaching without think too much about how to teach as my children were too young.

Yes. I had the qualification for the first time and I did not get promotion finally. I had been sad for a while and came back to be normal. I thought that maybe I still needed to work hard.

For the recent ten years, I have been the group leader and head teacher. Therefore, my workload has been much heavier. Currently, it is the heaviest.

I followed the advice of my parents: girls should still find a stable job.

Indeed, my mother has a great influence on me. She taught me that I should first think about what I did wrong when encountering difficulties.

After I had children, I could empathize with those students with learning difficulties and began to pay attention to them. Every time my child is praised by the teacher, or wins a small red flower, he is so happy.

Students’ influence

Influence of family members
