# Supplementary material for: An investigation of English language teachers’ motivation from an ecological perspective: A case study from mainland China
Source: PLoS One. 2025 Apr 29;20(4):e0321139. doi: 10.1371/journal.pone.0321139 (PMC12040097; doi:10.1371/journal.pone.0321139)
Supplement: S1 Data — (ZIP) [file pone.0321139.s001.zip › data analysis results/Lily's summary/LiLy' summary1.docx]

**Lily’s diagram 1**

I don't want to be a teacher, but when I became a teacher, I adapt to it quickly. I can find pleasures in it, and I did my work seriously

No, I liked Chinese very much and wanted to be a reporter or a writer. My teacher suggested that I could major in English and I could do many other jobs, such as being a journalist.

At that time, there was an urgent shortage of teachers, so I studied the English education.

I want to be a writer or a journalist. These jobs were challenging. I am extroverted and these jobs could enable me to have more experiences. There are few opportunities to see the outside world if I become a teacher.

The local education bureau selected teachers for high schools, and I became a high school teacher.

At that time, there was an urgent shortage of teachers in high school, which was also an occasional chance. I would like to have a try if I had the chance.

I had been a middle school teacher for three years.

Students in the middle school, especially it is a rural middle school, had few opportunities to expose to English since their childhood. Therefore, they learned English completely from scratch. They had difficulties in learning English and I had difficulties in teaching them. But students studied hard.

I followed the advice of my parents: girls should still find a stable job.

Unwilling to be a teacher

At first, I was reluctant to accept my identity as a teacher. However, I was adaptable. When I was a middle school teacher, I did my best. But I had been preparing the postgraduate entrance exam and wanted to leave this place. I was learning while teaching. However, my grades were not high and I was at the age for marriage. I had to stay there to teach.

Bein**g** a middle school English teacher in a village school

Choosing to major in English

Selection of high school teachers
