# Supplementary material for: An investigation of English language teachers’ motivation from an ecological perspective: A case study from mainland China
Source: PLoS One. 2025 Apr 29;20(4):e0321139. doi: 10.1371/journal.pone.0321139 (PMC12040097; doi:10.1371/journal.pone.0321139)
Supplement: S1 Data — (ZIP) [file pone.0321139.s001.zip › data analysis results/Lily's summary/LiLy' summary2.docx]

**Lily’s diagram 2**

When we talked about poetry during another wave of the pandemic, I wanted to put the students at ease and I asked them to go to the campus, getting close to the nature. I felt great. In addition, when explaining some knowledge points, I add some topics that are close to the real life for students. Therefore, the classroom teaching is not boring and students can understand more information. For example, tomorrow is Mother’s Day and I can use this as the leading topic to take resonance with students. I also relate my teaching with news and some small things in life.

Sometimes, they expand and teach too much of one knowledge point. For example, when they teach the phrase pick up, they may introduce seven or eight meanings of it. I prefer to introduce five of them, not introducing too much! Most of my colleagues would like to introduce words first but I prefer to explain words in its context. There are some changes of the words because of the person and tense. I do not like to talk about new words in isolation. I imitated others teaching methods first and then I found my teaching style.

Nearly ten years, no matter what level of students I meet, I rarely complain. Even when I teach less successful students, I focus on their advantages. In the process of reading their compositions, I mark out their good sentences.

I do not think about how to change others, but explore the reasons behind the phenomenon. Then I adjust myself and change my strategies in time. This is the most important. Complaining doesn't work and make you in a bad mood.

They should experience the use of language with their heart. Students commented that the highest frequency sentence I said was "Learn by heart".

The ought-to teacher self

But now I thought that I can achieve my purpose if my students’ grades are good and they can be admitted by their ideal universities. For students in backward areas, they have no other choice but go to the university to have a bright future. The university entrance examination can change their fate.

The rank of my students’ average grade should be among the top three.

I'm outgoing.

I am extroverted.

I am active and open. I am not as hardworking as other colleagues, although my grades in class are good

Tag: I think your emotional management abilities are outstanding.

I should be optimistic and learn to accept it. Therefore, I don't struggle with many things.

First, they may not encounter some of the knowledge. In addition, they may have stronger ability to accept more complicated knowledge based on their in-depth understandings of the basic knowledge. On the contrary, if students are given too many grammatical rules at one time, they feel lost. They may even fail to master the most basic and vital knowledge points. I believe that when they master basic knowledge, they are more likely to understand difficult knowledge. I try to something like this.

Lily’s personality

Teaching beliefs and methods
