# Supplementary material for: An investigation of English language teachers’ motivation from an ecological perspective: A case study from mainland China
Source: PLoS One. 2025 Apr 29;20(4):e0321139. doi: 10.1371/journal.pone.0321139 (PMC12040097; doi:10.1371/journal.pone.0321139)
Supplement: S1 Data — (ZIP) [file pone.0321139.s001.zip › data analysis results/Lily's summary/LiLy' summary3.docx]

**Lily’s diagram 3**

I want to teach students with the simplest method, which can be accepted by them easily and quickly. Therefore, they can benefit the most. In the process of my teaching, I continued to adjust my teaching method.

I just wanted to do my best and gave my students as much knowledge as possible. I wanted to get down to working.

To summarize, I initially want to teach them much more knowledge

I want to focus more on students with learning difficulties. How can I help those students have some interest and confidence in their study or life. they suffer more than good students. They're doing things they're not good at and they're not willing to do. Every subject is a challenge for them. They sit in classrooms where other students can learn, but they are unable to. It is a kind of psychological suffering for them.

Students learned individual knowledge points and then how can they find relations between those individual knowledge points and used them together to solve problems. In my routine teaching, I paid attention to the use of scenes, and gradually students had changes. Generally, such students were relatively simple and straightforward. With this awareness, they not only performed better in exams, but also changed in the process of communicating with teachers.

In addition, the knowledge in middle school was easy while that in high school was more difficult. Moreover, high school teaching was closely related with the university entrance exam. Therefore, teaching in middle school was different from that in middle school.

At the beginning, I was not confident. I cannot teach students well. I overestimated students’ language proficiency and learning abilities. Students were not happy and I was not happy.

I made simple problems complicated. In addition, I was unclear about complicated problems. My teaching was not effective.

Making students feel happy is more important than asking them to learn more knowledge. I paid attention to students' psychology before I was a head teacher.

My English teacher in the second year of high school had a great influence on me. The English teacher was very optimistic. I was also influenced by this English teacher and She liked to tell us about the interesting things happened outside world. I like it very much.

My high school English teacher and my mother exerted big influences on my style of dealing with people

My head teacher in the third year of the high school, taught math. My math was not good and I lacked confidence. I have a feeling for math in the second semester of grade three, I told the teacher that please do not laugh at me as I wanted to ask a very simple question

I thought that students in high schools studied harder as they were selected by the high school entrance exam. But I was wrong. They were older and were also picky about their teachers. Students had to get used to their teachers. Meanwhile, teachers had to get used to students at different levels with different personality.

Difficulties of being a high school teacher

Ideal teacher selves

Learning experience
