# Supplementary material for: An investigation of English language teachers’ motivation from an ecological perspective: A case study from mainland China
Source: PLoS One. 2025 Apr 29;20(4):e0321139. doi: 10.1371/journal.pone.0321139 (PMC12040097; doi:10.1371/journal.pone.0321139)
Supplement: S1 Data — (ZIP) [file pone.0321139.s001.zip › data analysis results/Lily's summary/LiLy' summary4.docx]

**Lily’s diagram 4**

My colleague's sister also more than once said that some of my ideas and practices are very good. However, I need a secretary to help me record them all. She said that I was too lazy. I need to organize my practices and ideas well. They are better than the content of many books on the market.

Yes, I am the group leader. I don't want to be the best, but I don't want to be the worst either.

I have been the leader for the joint lesson planning group for ten years. I didn't want to be the team leader at first, but I was assigned to be that. I was criticized by the head master that my mind was not broader enough and my work plan was not systematic.

It is good in other people’s eyes. My students’ grades rank relatively top.

For the recent ten years, I have been the head teacher, and have had more in-depth contact with students. Previously, I only considered students' English results. But after I became the head teacher, I consider things more comprehensively.

I have kept learning new knowledge. I always want to learn more. I want to grow up and become better. Meanwhile I want students become better with my help.

Meanwhile, I recently began to learn the courses of being a home educator.

Later, after I became a high school teacher, I went out to learn something new when I had the opportunity. After being a head teacher, I listened to lectures and read books. Some time ago, I always read the website of bright educator. Sometimes I felt that it was difficult to communicate with students and my children. I went to learn to be a family educator and some knowledge of psychological education.

Keeping learning all the time

Current teacher self

Being a leader of the joint lesson planning group

Being a head teacher

I am a teacher and I want to try my best to do a good job.

Although there were a variety of objective factors, I intend to attribute to my not good enough performance. I need to concentrate on my work and continue.

Although I didn't have the chance to see the outside world. Being a teacher is a job that shapes people. As I get older and more conscious, I think this job suits me well.

Attitudes towards the job

I do not want to let myself become a tired person psychologically. Physical tiredness is acceptable. My colleagues say that I am always happy even though I am so busy.

I don't want to be the teacher who hates by students.

The type of teacher that students hate is the one who is extremely distant from the students. These teachers do not understand students as they think things from their perspective.

Feared teacher self
