# Supplementary material for: An investigation of English language teachers’ motivation from an ecological perspective: A case study from mainland China
Source: PLoS One. 2025 Apr 29;20(4):e0321139. doi: 10.1371/journal.pone.0321139 (PMC12040097; doi:10.1371/journal.pone.0321139)
Supplement: S1 Data — (ZIP) [file pone.0321139.s001.zip › data analysis results/Lisa's summary/Lisa's summary1.docx]

**Lisa’s diagram 2**

I was influenced by others. Both my teachers and parents believed that studying English would help me have a better future than studying Chinese and history.

After graduation, I was assigned to be a high school teacher in my hometown. At that time, I was not allowed to change my work.

The researcher：Did you want to be an English teacher before you went to college?

Lisa：No, I did not think about these at that time. My score of the university entrance exam reached the requirement of this school.

I think language learning requires people’s divergent thinking, and I do not have advantages in this aspect.

The researcher：Are you interested in learning English?

Lisa：I was not really interested in it, but I can learn it. When I got to the university, I didn't think I was fit to learn English.

Because the knowledge in middle school and high school was simple, I did not feel that I had no interest in that or it was difficult to learn. However, when I came to the university, the content was difficult, and my learning progress was not smooth.

Learning experiences

The choice of English major
