# Supplementary material for: An investigation of English language teachers’ motivation from an ecological perspective: A case study from mainland China
Source: PLoS One. 2025 Apr 29;20(4):e0321139. doi: 10.1371/journal.pone.0321139 (PMC12040097; doi:10.1371/journal.pone.0321139)
Supplement: S1 Data — (ZIP) [file pone.0321139.s001.zip › data analysis results/Lisa's summary/Lisa's summary2.docx]

**Lisa’s diagram 2**

This was a great challenge for me. I was not good at getting along with others and I did not like communicating with students and colleagues. I was unable to have a close relation with them within a short time.

I don't think my personality fit me for the job. I used to think so, especially when I first started teaching.

I am the kind of person who is willing to do things well if I have to do it. It does not matter whether it's something I'm good at or not.

The Tag: If you had the chance, would you have changed your career?

Lisa：Yes

I like teaching classes now. When I first started, I didn't like going to teach and interact with students, but now I think it's fine. Maybe I'm just used to it.

Although I enjoy my job only moderately, but I spend most of my time at work.

I didn't want to communicate with people, but I still put most of my time into my work. I want to do the job well.

In the past, I was reluctant to participate in discussions about teaching in the office. I didn't think there was the need to discuss with other colleagues about the teaching. I was stubborn in believing that students can make their own progresses as long as both teachers teach carefully and students learn carefully.

The Tag: Are you uncomfortable with the questions I'm asking you?

Lisa：No. I have some changes now.

he Tag: I would like to ask your opinions first. In the thesis, your teaching experience will be reported, which is not used to be judged but to provide others some references.

Lisa: Yes, no problem.

The main purpose of the high school education, especially in a small county like this, is to improve students' grades. Schools in big cities may have richer goals for cultivating students. In this small county, students’ grade is the only aspect for assessment.

The focus of the school is students’ grades.

The Tag: So your goal at this stage is to raise the rank of the average score of students you are teaching to a medium level. Do you set a goal like this for each semester, which is related to the average score of the students?

Lisa：No. I have this kind of goal when I am serious. Sometimes, I feel I can change nothing.

Lisa’ feeling for being interviewed

Personality

Attitudes towards the job
