# Supplementary material for: An investigation of English language teachers’ motivation from an ecological perspective: A case study from mainland China
Source: PLoS One. 2025 Apr 29;20(4):e0321139. doi: 10.1371/journal.pone.0321139 (PMC12040097; doi:10.1371/journal.pone.0321139)
Supplement: S1 Data — (ZIP) [file pone.0321139.s001.zip › data analysis results/Lisa's summary/Lisa's summary3.docx]

**Lisa’s diagram 3**

I was stubborn and believed that the knowledge of English for high school students was easy. I kept doing what I believed was right. However, I gradually realized the inadequacy of my teaching.

When I first started teaching, I used the cramming method of teaching and I talked for most of the time. At first, because of the difference between my own perception and that of the students, I did not realize that the students needed to listen to teachers’ explanation of knowledge points. Later, when I realized the problem, I understood that some knowledge points needed to be taught systematically. Then, I realized that the autonomy power should be given to the students, so that they can find problems by reading and learning. Their problems should be the focus of teachers’ explanation and analysis.

My idea at that time was very simple. I thought it was easy for me to be a high school teacher after I graduated from the university.

The teacher talks and the students take notes. At that time, I thought that I can teach effectively as long as I know and understand that knowledge. After one or two years, I realized that there were gaps of information and knowledge between teachers and students.

I lost my confidence and did not believe that I could make any achievements.

The Tag：Could you tell more about the specific difficulties?

Lisa：I was not good at communicating with students. Some of the knowledge is very easy and I think they did not need detailed explanation. However, students can learn it well only by teachers’ detailed explanation.

I was willing to help students have high grades but I failed to. I was so depressed and I believed that I needed to change my teaching methods

The researcher：Have you encountered any difficulties since you started teaching?

Lisa：It's very difficult. Teaching and learning are two very different things. Learning depends on my own efforts. If I study hard, I can learn well. But for teaching, I worked very hard but I did not teach well.

I've been unsatisfied with my teaching.

There's still a lot to be unsatisfied with. It's still a long way between I and an excellent teacher.

The Tag：What are you most dissatisfied with yourself now?

Lisa：That is my influence on the students and the students' grades cannot reach my satisfaction.

The Tag：How about your teaching abilities?

Lisa：I am not satisfied with that either.

Difficulties during the teaching career

Teaching beliefs and methods

Current teacher self
