# Supplementary material for: An investigation of English language teachers’ motivation from an ecological perspective: A case study from mainland China
Source: PLoS One. 2025 Apr 29;20(4):e0321139. doi: 10.1371/journal.pone.0321139 (PMC12040097; doi:10.1371/journal.pone.0321139)
Supplement: S1 Data — (ZIP) [file pone.0321139.s001.zip › data analysis results/Lisa's summary/Lisa's summary4.docx]

**Lisa’s diagram 4**

I think that the whole school environment is getting better.

If the school can be managed well, teachers’ motivation can be stimulated to the largest extent. The whole atmosphere of the school has been changed a lot.

For example, especially at this stage, the new headmaster cares for teachers in all aspects such as their lives and the management of teaching activities, making teachers feel warm. He thinks for teachers with more consideration and care, and manages teaching activities more carefully. He makes teachers feel warm and passionate from the bottom of their heart. They do a good job to solve students' problems, setting us teachers good examples. For example, when it rains heavily, the headmaster and other leaders rush to the school to check the safety problems. What they do touches teachers and we become more united.

The quality of teachers is improving all the time. The most obvious thing is that the teachers' education level has improved. When I first started as a teacher, most of other teachers graduated from junior colleges and few of them had bachelor degrees. There are now many teachers who have master degrees. In addition, these young teachers had grown up in a better environment, receiving a better level of education. Therefore, their overall quality is high and give others a good impression. Some of them are born to be teachers. At their initial stage as a teacher, they perform better than those experienced teachers in terms of controlling the teaching pace and communicating with students.

At the beginning, I think teaching is that teachers teach their own knowledge to the students. And then I realized that the teaching methods and the relationship between the students and teachers influence significantly.

When I meet students with good grades, I feel that my work is valuable. When I meet students with poor grades, I feel that my work is meaningless.

The Tag：If you encounter students who are not doing well, your motivation to teach will be reduced.

Lisa：Yes, I then feel that my work is meaningless and not worth doing.

The Tag：Because of the poor attitude students have for learning?

Lisa：Yes. Students refused to learn no matter how hard I worked.

Tag：How about those students who did not learn well but had a serious attitude.

Lisa：There is hope for such students. As long as students keep learning, and teachers teach carefully, and constantly adjust the method, students can make progresses.

The Tag：Why did you change your thinking?

Lisa：Because of students’ comments. What impressed me was one thing that one of the students asked me a question, and I explained it in a few words and then left. Later, a colleague told me that the student said my explanation was too simple and he did not understand it. I realized there was something wrong with the way I was teaching.

The management of the school

Colleagues’ influence

Students’ influence
