# Supplementary material for: An investigation of English language teachers’ motivation from an ecological perspective: A case study from mainland China
Source: PLoS One. 2025 Apr 29;20(4):e0321139. doi: 10.1371/journal.pone.0321139 (PMC12040097; doi:10.1371/journal.pone.0321139)
Supplement: S1 Data — (ZIP) [file pone.0321139.s001.zip › data analysis results/Sophia' summary/Sophia's summary1.docx]

**Sophia’s diagram 1**

When I was in high school, I had an idea that I didn't want to be a teacher. Because being a teacher would have a great influence on students. If I didn't do it well, it might affect students' whole life or at least have an impact on their high school study. Teachers assume heavy responsibilities.

Maybe it is a coincidence. My grade of the university entrance exam was not good. I was enrolled by the normal junior college and then I continue my study in the university

But there were some difficulties. My spoken English was not good and I felt that my tongue was not flexible. At the beginning, my classmates were all similar in spoken English. But after a year or two, I found that others improved their spoken English a lot. At that time, the teacher asked us to record. Others imitation was very successful.

Then I continued my study in a normal university.

When I filled the application form for university, I didn't know much about it. I just wanted to be closer to home, so I applied for a local normal junior college.

I planned to go to back my home immediately after my graduation. My parents were quite liberal and didn't interfere my choice. It was because of my personality. I preferred to come back to my hometown. I was familiar with the local conditions and customs. I can communicate well with students. It was easier for me to get along well with others. When I was a university student in another city, there were many different living customs which I did not know much about them although they were in the same province. When I returned to my hometown, I felt a sense of security.

Tag: You learned English education?

Sophia: Yes. I had teaching certificate for English in middle school and high school.

The choice of majoring in English education

When I was in high school, my best subjects were English and Chinese. At that time, my best subject was Chinese, and I liked reading and writing. However, when I applied for the major, I did not choose Chinese. Because I felt that thinking too much made me sentimental, which was not good for myself. My English score was also good, so I chose English.

Unwilling to be a teacher initially

Performance of the university entrance exam

English learning experience

Being homesick
