# Supplementary material for: An investigation of English language teachers’ motivation from an ecological perspective: A case study from mainland China
Source: PLoS One. 2025 Apr 29;20(4):e0321139. doi: 10.1371/journal.pone.0321139 (PMC12040097; doi:10.1371/journal.pone.0321139)
Supplement: S1 Data — (ZIP) [file pone.0321139.s001.zip › data analysis results/Sophia' summary/Sophia's summary2.docx]

**Sophia’s diagram 2**

I've been working hard all the time, which I'm proud of. I have never slack off not matter what kind of classes I teacher, or whether I have professional title promotion. It does not have a big impact on me.

I work over timely to give students feedback in time.

Although the workload is heavy, I am satisfied.

Attitude towards the job

I'm not a very social person myself. I stayed at school most of the time.

It made me cry. I am more emotional and not a rational teacher.

Yes. I am always worried about something.

When I write a reflection journal, I mainly focus on problems but I do not intend to record what I do well.

Her personality

Students are different from year to year. The older I am, the bigger age difference between I and students. We become distant and not so close. Now I don't know much about students and my understanding about them are from other resources. Even if I talked with some of them face to face, it is difficult for me to find a suitable breakthrough point to open students' hearts and establish a more intimate connection.

I am a little crystallized in my thinking. Sometimes

I dare not to try something new because I am afraid of students' opposition or that the effect is not good. I do not rich experience of being a head teacher and do not have a broader eyesight.

But the new curriculum standard now has a practical problem. The completing of such a reading class needs to build on the basis of students having sufficient time to make full preparation, such as previewing and looking at reference materials in advance. Otherwise, tasks of the course cannot be completed. In the training of the summer vacation, the expert suggested that we needed two classes to complete the reading course. But in the practice, we cannot operate like this. So I am confused.

Some students write to me and tell me that they have no motivation to learn. But I felt that my words were not persuasive when I gave guidance to them. My words failed to touch and motivate them.

In addition, there is a question named the continuation writing. I was excited when I first learned that there was such a continuation writing.

However, I found that students' ideas were various, and they could not go on the right track. I think the clue for writing is clear and I cannot understand why students think in different ways. They have various problems because of their limited experiences and thinking and reading abilities.

Moreover, I failed to get the highest mark in the exam sited together by teachers and students. Other colleagues expect that I should win the first place.

My students’ grades were outstanding, the top one. But now other teachers’ students surpass my students’ grade

Difficulties in the current stage
