# Supplementary material for: An investigation of English language teachers’ motivation from an ecological perspective: A case study from mainland China
Source: PLoS One. 2025 Apr 29;20(4):e0321139. doi: 10.1371/journal.pone.0321139 (PMC12040097; doi:10.1371/journal.pone.0321139)
Supplement: S1 Data — (ZIP) [file pone.0321139.s001.zip › data analysis results/Sophia' summary/Sophia's summary3.docx]

**Sophia’s diagram 3**

However, he is very strict with his students. Such rigor makes students feel no love.

He criticizes students harshly. He only cares about himself and ignores others' feelings. His way of doing things was too extreme that he scolds students when they fail to submit their homework on time. Students are afraid of him.

The leaders praise the teachers whose students’ grades are good. Teachers whose students’ grades are not good may be required to write down their teaching reflection and submit that to the leader.

There is another problem that if teachers do not teach seriously and students’ grades are good enough, teachers are not allowed to follow students into higher grades. The leader of teachers of each grade has the right to decide whether there is a position for teachers like that.

Feared teacher self

The global environment has changed and there are various evaluations and appraisals. The recent two presidents emphasize students’ grades prominently.

Students’ grade is everything.

I want them to be No.1.

I think it should be a teacher with a good command of English professional knowledge

I think have sufficient professional knowledge. But I think I need to learn more and improve myself.

At the beginning, the exam focus is grammar rules.

After the reform of college entrance examination, teachers are required to have more knowledge.

After the reform of the university exam, the new focus is sentence structure. There are more questions about long and difficult sentences. In addition, the difficulty has been increased.

When students’ response was overwhelming, I feel a sense of accomplishment. On the contrary, I feel bad when students’ response is not like my expectation.

When I pay attention to the effect of each class, students’ grades can be improved.

I mainly focus on the teaching effect of each class.

Try to be as egalitarian as possible.

The teacher cared about students with good grades and ignored students with poor grades. Once I was ranked low because the score of one subject was not included in the total score. The teacher did not pay attention to me. However, I ranked the top five next time and the teacher began to care about me. The teacher asked me to answer questions in class. I think this was not good.

Tag：What do you mean by wanting to be recognized by students? Such as the teacher is serious and responsible?

Sophia：Yes. It also means that students think that I am excellent and teach well. They like my class.

Tag：What do you mean by teaching well？

Sophia：For example, students can understand new knowledge points with my explanation. They can truly understand that and do practice. Understanding new knowledge points is a very basic level. The second stage is to do exercise right. The third stage is to gain an in-depth understanding and can make an analogy to them. Then students can figure out different types of questions related to these knowledge points. This is the level of in-depth understanding and application.

The ought to teacher self

Ideal teacher selves
