# Supplementary material for: An investigation of English language teachers’ motivation from an ecological perspective: A case study from mainland China
Source: PLoS One. 2025 Apr 29;20(4):e0321139. doi: 10.1371/journal.pone.0321139 (PMC12040097; doi:10.1371/journal.pone.0321139)
Supplement: S1 Data — (ZIP) [file pone.0321139.s001.zip › data analysis results/Sophia' summary/Sophia's summary4.docx]

**Sophia’s diagram 4**

The feedback from the students was that they recognized and liked me very much. They thought that I was serious and responsible. But their former teacher was not serious.

In the first year, I was lucky. My class performance was very good. One class ranked the first and another one was the third.

I do not rich experience of being a head teacher and do not have a broader eyesight.

I am a little crystallized in my thinking.

I think that some methods may have a big impact on some students’ learning from the whole aspect. I occasionally try new teaching methods. I think I should try this. If you look at the courses of famous teachers, you can find that their teaching methods are very flexible. The students are happy, and the cooperate with their teachers well. ……However, the time of the class is limited.

Some teachers ask students to study the first three required course books, and then ask students to memorize words and do exercises. I don't think it's good. But students of these teachers have good grades.

They just find out phrases and words to ask students to memorize, I think these are meaningless.

In my opinion, language learning is not only about knowledge points but also about the cultivation of thinking.

I always feel anxious. When I was the head teacher, I always worried something unexpected would happen. I felt very tired. It may have relations with my problem-solving ability and mood

At that time, I always looked at the problems of my students and was in a bad mood.

There were good memories and touching moments when I was with my students. I did not want that there were some problems for my class. I was an idealist.

Current teacher self

Actually it is very useful. the purpose of memory is to make links between individual knowledge points. Meanwhile, this can keep your mind active and enhance their ability to use language. In addition, students can strengthen their memory of words and phrases. This is more effective than recite words and phrases directly. Students should keep thinking and their mind active

The experience of being a head teacher

Teaching beliefs and methods
