# Supplementary material for: An investigation of English language teachers’ motivation from an ecological perspective: A case study from mainland China
Source: PLoS One. 2025 Apr 29;20(4):e0321139. doi: 10.1371/journal.pone.0321139 (PMC12040097; doi:10.1371/journal.pone.0321139)
Supplement: S1 Data — (ZIP) [file pone.0321139.s001.zip › data analysis results/Sophia' summary/Sophia's summary5.docx]

**Sophia’s diagram 4**

I feel motivated when students called me "teacher Sophia" and when they showed their gratitude after I answered their questions. I am happy when I see their progress.

There are so many serious, studious, and dedicated students in the class. They are my daily fuel!

Experienced teachers have a good command of these grammar rules and I asked their suggestions when I had difficulties.

No. we are harmonious. We think others are excellent.

Recently, I have a strong sense of accomplishment when I teach in the good class.

When I teach the class with lower grades, the students are more convinced of what I teach. But the students in the other class who are doing better feel that they don't need to remember what I'm saying, because they basically know it. But it goes smoothly in the good class if we talk about something difficult.

They do not care about Chinese and English. I get angry when they do not finish English homework in time. I've tried to talk to students who don't hand in their homework. But it doesn't work.

There are a few students do not change no matter how hard I try.

Students are different from year to year. The older I am, the bigger age difference between I and students. We become distant and not so close. Now I don't know much about students and my understanding about them are from other resources. Even if I talked with some of them face to face, it is difficult for me to find a suitable breakthrough point to open students' hearts and establish a more intimate connection.

The influence of students

The influence of colleagues

My parents are farmers and they want to do well whatever they do. They think that the serious working attitude is even more important when I work for the government. And they think it's important to be a teacher.

My husband is very busy with his work. Our parents in the family are in the countryside, and we have to take care of the child by ourselves. We are too busy to take good care of and educate our child well. We spend most of our time in working.

Influence of family members

The environment not only affects me, but also affects my colleagues. Colleagues who had been lazy and did not pay attention to their students’ grades have changed.

I want to obtain the honor title of the school. I've been working hard, but I haven't won many honors.

The current evaluation method is not fair for my classes. Students’ grades are already high and there is little space for their improvement. I can understand this as this does not target on me.

I used to think that fame and fortune were not important. But now I think it is a kind of pursuit and a sense of honor. we all want to go to receive the prize at the annual Teachers' Day commendation meeting. It is a kind of recognition of their work.

The requirements for teachers and students are high. I am busier.

At that time, the environment changed. Quality-oriented education was advocated. There were no such high requirements for teachers to improve students’ grades. I did not have pressure for this aspect.

The global environment has changed and there are various evaluations and appraisals. The recent two presidents emphasize students’ grades prominently.

The management of the school
