# Supplementary material for: An investigation of English language teachers’ motivation from an ecological perspective: A case study from mainland China
Source: PLoS One. 2025 Apr 29;20(4):e0321139. doi: 10.1371/journal.pone.0321139 (PMC12040097; doi:10.1371/journal.pone.0321139)
Supplement: S1 Data — (ZIP) [file pone.0321139.s001.zip › data analysis results/Wynne's summary/Wynne's sumamry 2.docx]

**Wynne’s diagram 2**

I think it's because of my personality, which is not very good. I used to think that students didn't do well in exams because they didn't study hard. But now I think it's mainly the teachers who are responsible for the bad results. When I think that in this way, I am not angry any more.

I am not willing to participate social activities to some extent, which means I do not want to contact with other adults too much.

As I'm not very good at dealing relationships with other adults, it is easiest and best for me to get along with students. It is the best and most relaxing to work with vibrant children. It is much better than getting along with adults.

I have become more and more fond of this job in recent years. In summary, I worked for students’ grades previously. However, my well-being has been promoted for the last few years.

I like my work.

When I am working, I feel happy. Teachers talk and laugh in the office. I can feel happiness in work and at home. They are different. I don’t think my job is boring. I do not have negative emotions except for feeling tired sometimes. I try my best to work. Everyone says that being a teacher is a matter of conscience. I don't think it is necessary to rise it to this height or be morally bound. My idea is simple. It is fine with me as long as I work hard.

The researcher：What are your specific requirements?

Wynne: Their ranking should be around the middle. Or at least one class is doing well.

I worked for students’ grades previously.

I think all high school teachers care about their students' grades. I was lucky at the beginning. The students’ grades of one of my classes got the first place.

In the past, there were neither so many comparisons of scores nor such detailed examination analysis meetings. Sometimes teachers evaluated their students’ grades by themselves. In addition, they were not familiar with their own students’ and students’ scores of other teachers. At that time, the emphasis on scores was not so obvious.

I think that teaching should be like this. Teachers should teach what they know, help students improve their grades, and work carefully.

During these previous years, I did not feel happy in my teaching. I like it more and more over the years. I can enjoy my teaching and the good time spent with my students.

I mean that I neither like it or dislike it. It is only a job for me and I treat it seriously.

Attitudes towards the job
