# Supplementary material for: An investigation of English language teachers’ motivation from an ecological perspective: A case study from mainland China
Source: PLoS One. 2025 Apr 29;20(4):e0321139. doi: 10.1371/journal.pone.0321139 (PMC12040097; doi:10.1371/journal.pone.0321139)
Supplement: S1 Data — (ZIP) [file pone.0321139.s001.zip › data analysis results/Wynne's summary/Wynne's sumamry 3.docx]

**Wynne’s diagram 3**

I was courageous. I signed up for the public courses for many years as long as there was an opportunity. I polished my courses again and again in advance. Basically, by delivering public courses for many years, I gradually got ideas about how to give lectures. Until now, there are still some things of my courses I am not satisfied with.

Then you want to improve your teaching from two aspects. One is that you want to make your course clearer, such as appropriate teaching methods, clear teaching objectives and teaching steps; the other one is that you want to teach knowledge in a more logical and systematic way.

I don't want to be the teacher who has a particularly tense relationship with her students. Teachers exert a lasting impact on students no matter how much knowledge they teach students. Students may not be particularly outstanding or excellent in learning. I don't want to be the teacher that students hate in terms of life coaching (guidance) and teacher-student relationships. I don't want to be such a teacher because I have such kind of colleagues. I don't in favor of his or her ways of doing things even though he or she wants to do a good job and improve students' grades. Other colleagues also gave him suggestions that he should not always communicates with students in a scolding tone. But he does not change and is always unfriendly to his or her students. I think that he has many problems in dealing the relationship with students and students’ evaluation on him or her is not positive.

Previously, I thought high school students didn't have time to practice speaking. A few years ago, I thought that it would be a relatively advanced goal of teaching that students could apply the words, phrases and sentence patterns they learned into writing. But now I find that spoken English is the most difficult for students. Because students need to express their opinions in a short time. I still hope to seize some opportunities and time to ask students to practice oral English……. My current goal is to help students improve their writing and speaking.

I especially like teaching students how to write. I summed up a set of rules in the accumulation of writing materials and developing writing ideas. Later, my method has been promoted among the whole school. This really make me feel a great sense of fulfillment. Although I am very tired and need to spend a lot of time reading and commenting students' compositions, I am very happy to do that. I like to do it. I am also very happy When I see other colleagues are using my method.

Because students are good at learning English, there is an opportunity to help them improve their writing and speaking.

I have a student who entered a university in Beijing after graduating from this school. Once he sent a Wechat moment said that he felt that he learned fake English. His comments influenced me significantly.

The feared teacher self
