# Supplementary material for: An investigation of English language teachers’ motivation from an ecological perspective: A case study from mainland China
Source: PLoS One. 2025 Apr 29;20(4):e0321139. doi: 10.1371/journal.pone.0321139 (PMC12040097; doi:10.1371/journal.pone.0321139)
Supplement: S1 Data — (ZIP) [file pone.0321139.s001.zip › data analysis results/Wynne's summary/Wynne's sumamry 4.docx]

**Wynne’s diagram 4**

To be honest, teaching should be a collective activity. In this way, teachers can grow up together.

The researcher：Do you explain knowledge points in detail?

Wynne: No, we find phrases and sentence patterns together. For those difficult sentence patterns, I explain a little bit. Most of the time are spent for students to practice them. It's more important to let students know how to use them. I do not think it is efficient to teach students a great amount of knowledge points without providing them chances to use the language.

For example, the reading class basically consists of two lessons. The first lesson is comprehending the main content of the passage, and the second lesson is teaching grammatical points. Some teachers prefer to work through both steps in one lesson, while others prefer to teach the essay by paragraphs. I personally prefer to have some time for students to practice their oral and written English. After the first class, I ask students to practice their oral English. After the second class, I ask students to write short essays by compiling learned knowledge points.

I encourage them to speak more English and I tell them to realize the communication function of English is one of the important leaning goals. Moreover, oral English practice can help them organize their English expressions more quickly. I ask my students to write three English diaries for every week.

I hope that their learning of English is not solely for the university entrance exam but for communications with foreigners. When it is needed, they can use English to communicate freely. At least they have this kind of courage and can say several sentences in English.

I now teach more systematically with clear purpose. When I teach classes, I can take into account all aspects of listening, speaking, reading and writing. Moreover, I also take into account the situation of students. Therefore, I consider knowledge more systematically and comprehensively.

At my age, I learn that I can't solve anything by worrying yourself. It is also true in my ordinary life that emotions cannot help solve the problem. I should figure out methods to solve problems.

The continuation writing is like a complete story, which can reflect students' values. We guide students to form positive ideas. This kind of writing can reflect students’ comprehensive abilities to solve problems and their common sense of life. I like this kind of writing as students should have abilities of expressing, reading and understanding.
