# Supplementary material for: An investigation of English language teachers’ motivation from an ecological perspective: A case study from mainland China
Source: PLoS One. 2025 Apr 29;20(4):e0321139. doi: 10.1371/journal.pone.0321139 (PMC12040097; doi:10.1371/journal.pone.0321139)
Supplement: S1 Data — (ZIP) [file pone.0321139.s001.zip › data analysis results/Wynne's summary/Wynne's summary 5.docx]

**Wynne’s diagram 5**

In the past, there were neither so many comparisons of scores nor such detailed examination analysis meetings. Sometimes teachers evaluated their students’ grades by themselves. In addition, they were not familiar with their own students’ and students’ scores of other teachers. At that time, the emphasis on scores was not so obvious.

Since the new principal come, we are positive. He himself also has the power of a good example. He is particularly eager to improve the school as a whole. His hard work infects us.

The school tends to give new teachers more chances to help them improve. I am especially grateful for these opportunities and valuable suggestions from my colleagues to help me grow gradually.

The relationship between colleagues become more harmonious.

We are discussing how to deliver courses in a better way and how to improve students' grades.

More and more schools in other places came to enroll excellent graduates from high schools. Therefore, most of the students who stayed were not good at learning. Teachers were worried about this and only a few graduates could be enrolled by universities. This is one of the reasons for teachers’ demotivation in the past.

Yes, it is also because of the two leaders of the education institution, who have done a lot of work to reverse the situation of losing excellent students and retain most of the high-quality students.

Teachers are highly motivated. They all try their best to achieve those clear goals. Everyone emphasizes student’ grades. Moreover, the accumulation of work experience is important. The collective preparation and discussion for lessons are really helpful. This is the collective wisdom. All these are helpful to the overall performance improvement.

There have been rising requirements of students’ grades for teachers to achieve these recent years. For four to five years, we were confused and not motivated so much as students’ grades as a whole was not good. It is quite different now as we all have clear goals for students’ grades.

At that time, we did prepare courses collectively and each teacher designed his or her own courses based on individual’s own understandings.

Now there is a very good practice in schools, in which new teachers observe other teachers’ class and they also be observed by other teachers. After that, we discuss together and everyone can express their opinions.
