# Supplementary material for: An investigation of English language teachers’ motivation from an ecological perspective: A case study from mainland China
Source: PLoS One. 2025 Apr 29;20(4):e0321139. doi: 10.1371/journal.pone.0321139 (PMC12040097; doi:10.1371/journal.pone.0321139)
Supplement: S1 Data — (ZIP) [file pone.0321139.s001.zip › data analysis results/Wynne's summary/Wynne's summary 6.docx]

**Wynne’s diagram 6**

I always feel that I serve for students, who are bright, energetic, and simple.

Being a teacher is different. Students are living lives, having their own characteristics and advantages. Teachers can be true friends with students without the purpose to improve their grades.

when I see their growth and progress, I feel good; When I communicate with them, I feel good; When they have problems, they find me and want to get some suggestions, I feel good as they trust me.

Students are very simple and they always have new ideas, and represent a trend. Most of the time, I understand trendy things after my students explain it. The gain of being a teacher is not only limited to the improvement of students’ grades. It also includes various valuable feelings. Some graduated students ask me to go out to have fun with them. This is also a happiness for being a teacher.

That feeling has changed. I used to think of my communication with students as communication between peers. But now our relationship is the same as that between mother and son. Because my child will enter high school next year, I call my students "my baby". I Just change into a different identity.

I haven't really thought about that. The students are true to me, and I am true to the students. We trust each other. There are no principles. Some children don't like studying, but they are good in other aspects, such as love to communicate and chat.

I like talking with students. They would find me and talk with me as students trust me. Students are closer to me. Sometimes they would leave notes and snacks for me. Some boys would run from far away and hug me. I am touched by them. When I just graduated, I was not much older than my students. We have a lot to talk.

In addition, when I began to teach students of grade three, the school assigned me two science classes, in which students were with the solid foundation and the strongest learning ability.

I have grown significantly since I taught these excellent students.

I am very happy as I have a strong sense of happiness and fulfillment. I need to respond immediately and to prepare carefully as students usually ask all kinds of questions, a lot of them involving details.
