# Supplementary material for: An investigation of English language teachers’ motivation from an ecological perspective: A case study from mainland China
Source: PLoS One. 2025 Apr 29;20(4):e0321139. doi: 10.1371/journal.pone.0321139 (PMC12040097; doi:10.1371/journal.pone.0321139)
Supplement: S1 Data — (ZIP) [file pone.0321139.s001.zip › data analysis results/Wynne's summary/Wynne's summary1.docx]

**Wynne’s diagram 1**

The English teacher in my junior middle school was the only one who graduated from English major there, and the other teachers are teachers of general subjects, which means that they were not professional. The pronunciation of that teacher was authentic and his explanation of words collocation and grammar explanation was clear and orderly. I am especially grateful to him as he is my first English teacher and very professional. Hence, the foundation of my English knowledge was solid to some extent. There was also another English teacher used heuristic teaching methods, who was also very professional. Up to now I still remember how she taught me the rules of how to use leading words in attributive clauses. I think that the profession of a teacher has significant influence on students. There was also another teacher who focused on our understanding about texts. Therefore, she did not teach us lots of grammatical rules. I think that she wanted us to find some fun in our study and we all like her courses.

I thought that being an English teacher could provide me a chance to experience the outside world. I had no idea about being a high school English teacher. I could only know the job through my teacher. I was lucky that two of my head teachers in the high school were English teachers, and we got on well with each other. The first head teacher, like a loving mother, shared us important principle and delicious food. The second was like my friend, who was sincere and taught us singing and dancing. Therefore, I think teachers should be friends with students.

Two of my head teachers in the high school were English teachers. I had very good relationships with them. Although my English grades were not as high as those of my Chinese, I did not hate English. In addition, when I began to learn English in junior high school, my listening and speaking were not bad. I felt good then as my English grades were not low. I'm not a smart person. Therefore, if I wanted to make some progresses, I needed to pay great efforts. After I entered the university, my grades were not bad and I did not feel frustrated. I was happy when I studied there. Moreover, there were foreign teachers. I thought that I might also had chances to teach foreign children Chinese. That would be great. In summary, I both like English and Chinese very much.

Learning experience

The choice of English major

I was one of the last group members who were junior college graduates being teachers in this school.

The school was short of English teachers when I graduated in 2004…..I was relatively lucky as since then only graduates form universities could become a high school teacher.

I found that there was short of English teachers in this county. Therefore, it was easy for me to find a job if I chose English as my major. It was simple

The researcher: Did you study English in a normal University?

Wynne：Yes, my target was to be a teacher.

At that time, this high school and another high school were very short of English teachers. I was lucky to have a stable position in a public high school. Some of my other classmates went to private schools and then need to pass the exam of teachers’ recruitment for positions in public schools. For me, I only needed to take the exam and interview organized by this school. After that, I became a teacher here.

Participating in the selection process by this school
