# Supplementary figures and images for: An investigation of English language teachers’ motivation from an ecological perspective: A case study from mainland China
Source: PLoS One. 2025 Apr 29;20(4):e0321139. doi: 10.1371/journal.pone.0321139 (PMC12040097; doi:10.1371/journal.pone.0321139)

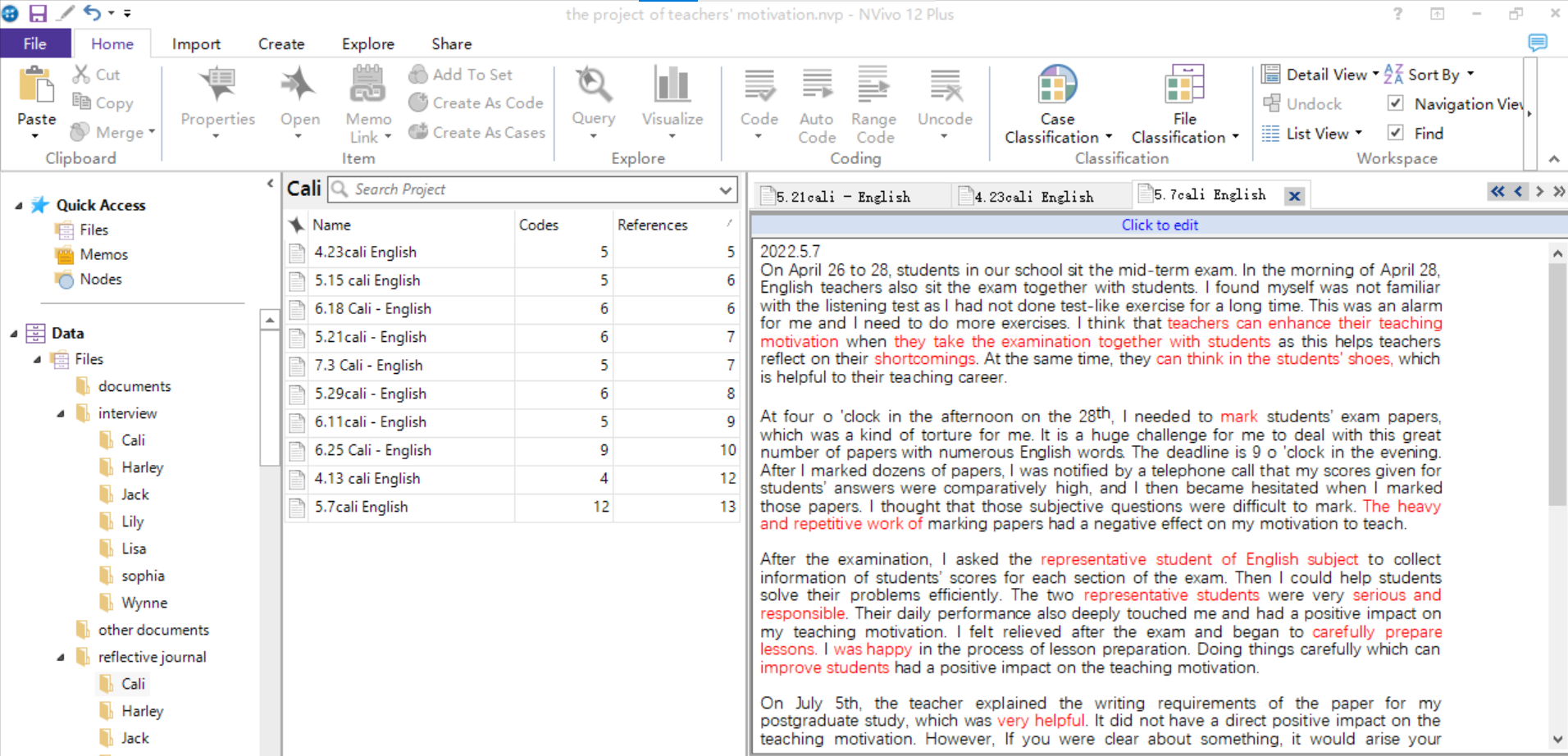

Supplement: S1 Data — (ZIP) [file pone.0321139.s001.zip › data analysis results/Cali‘s summary/Coding Cali's refelctive journal .png]

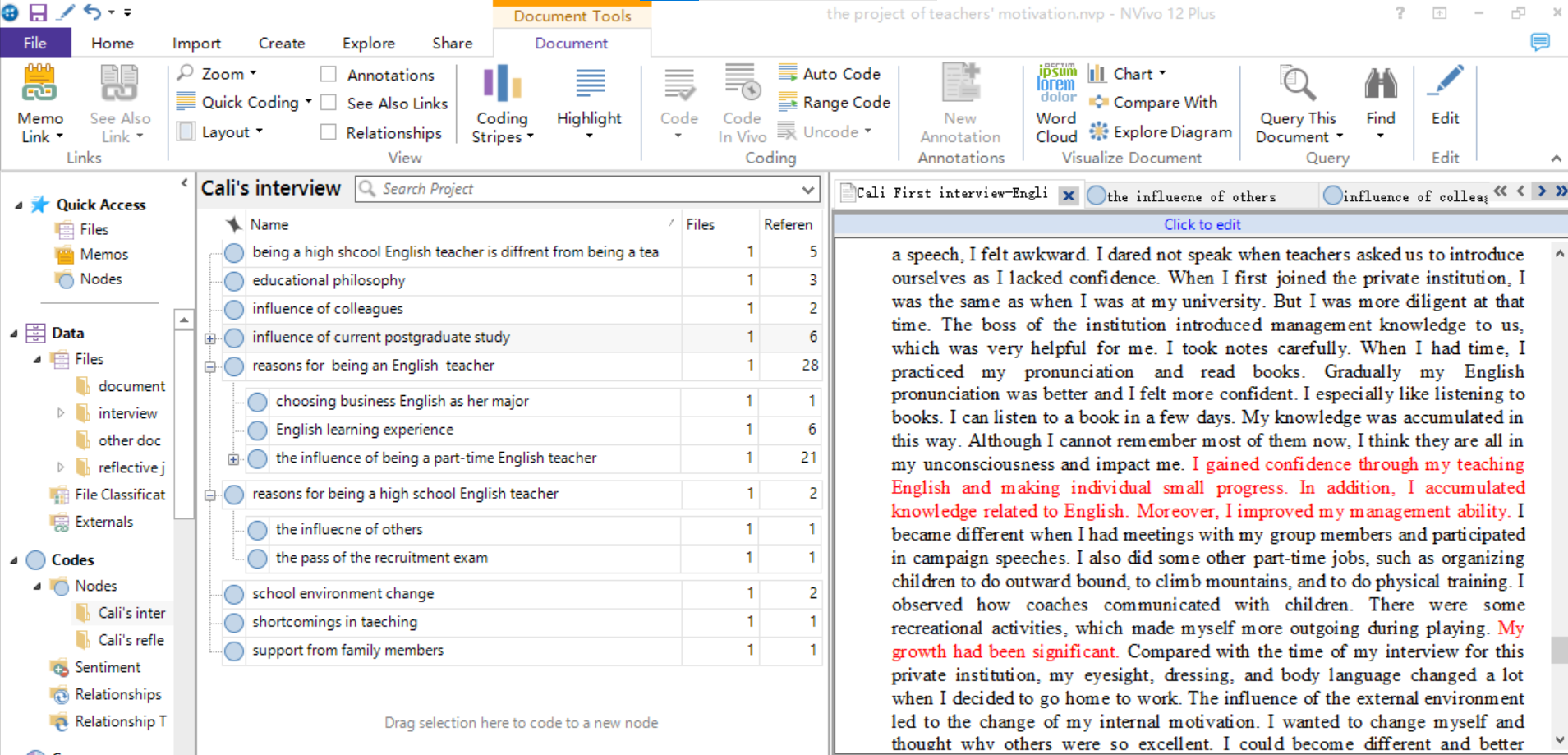

Supplement: S1 Data — (ZIP) [file pone.0321139.s001.zip › data analysis results/Cali‘s summary/The codes of Cali's first interview .png]

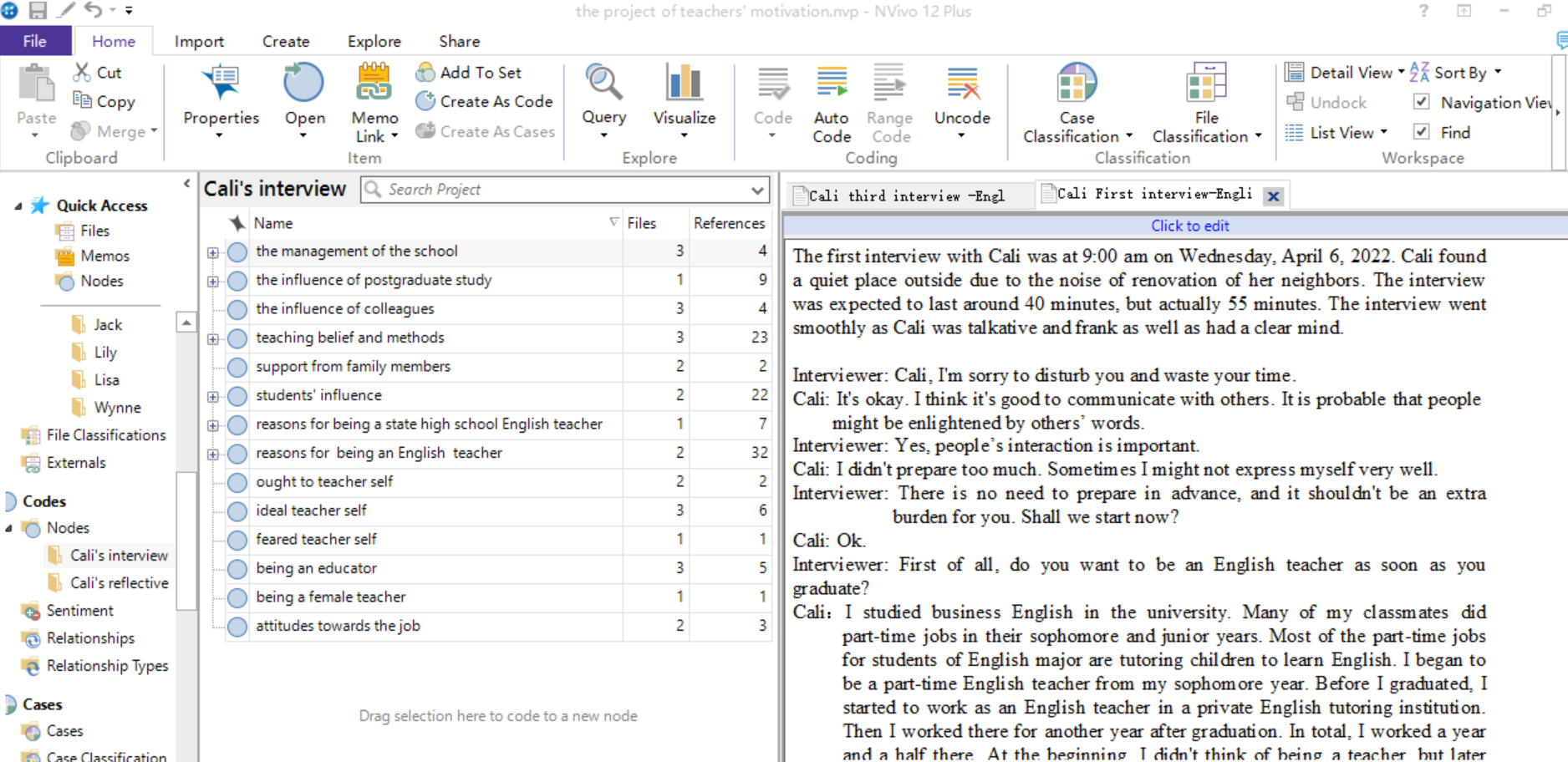

Supplement: S1 Data — (ZIP) [file pone.0321139.s001.zip › data analysis results/Cali‘s summary/The codes of Cali's interview.png]

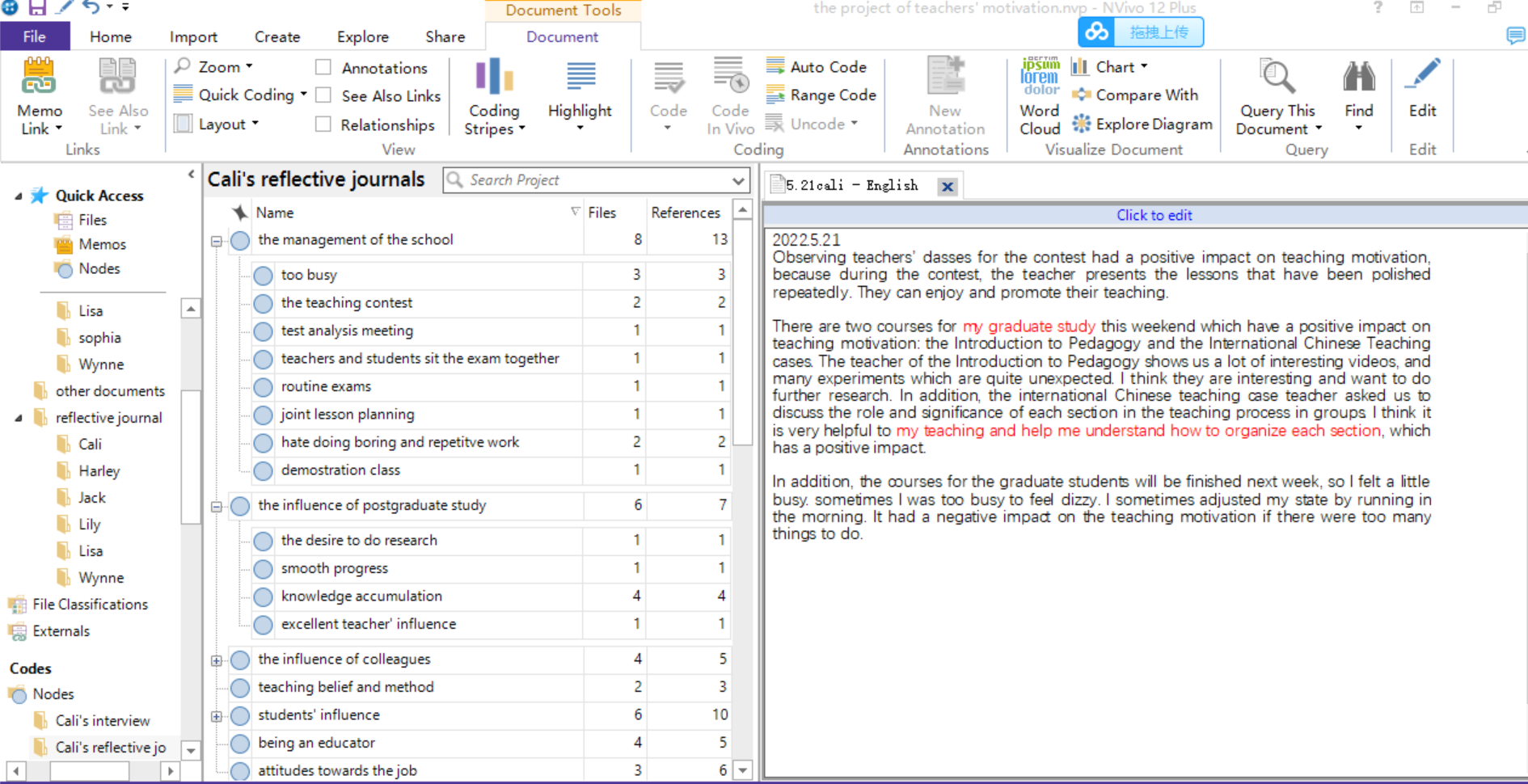

Supplement: S1 Data — (ZIP) [file pone.0321139.s001.zip › data analysis results/Cali‘s summary/The codes of Cali's refelctive journals .png]

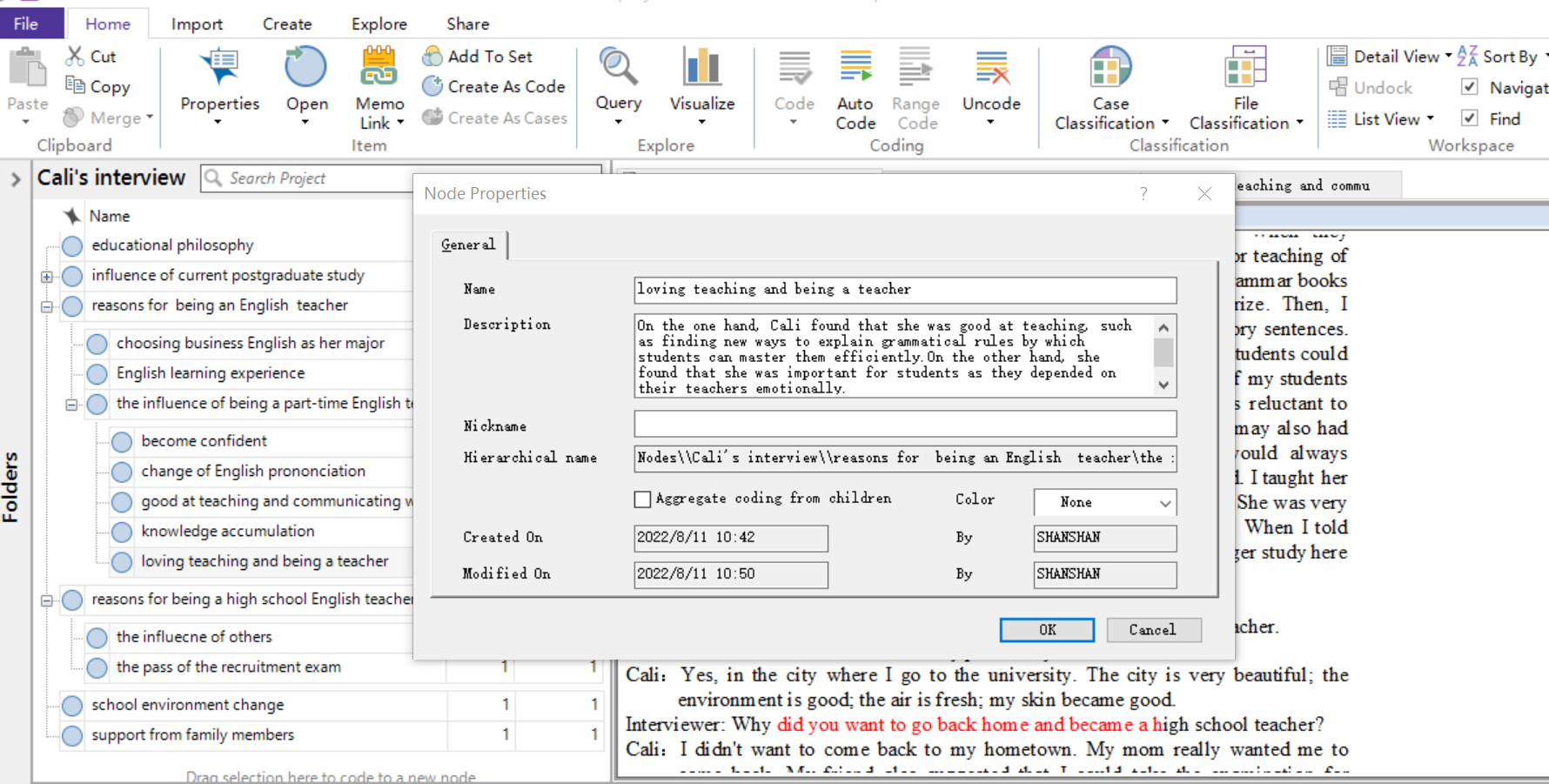

Supplement: S1 Data — (ZIP) [file pone.0321139.s001.zip › data analysis results/Cali‘s summary/making notes for the code .png]
